# Supplementary material for: Betulinic Acid-Nitrogen Heterocyclic Derivatives: Design, Synthesis, and Antitumor Evaluation in Vitro
Source: Molecules. 2020 Feb 20;25(4):948. doi: 10.3390/molecules25040948 (PMC7070564; doi:10.3390/molecules25040948)

Supplementary Material for compounds:

Compound 3

^{1}H-NMR spectra of Compound **3**

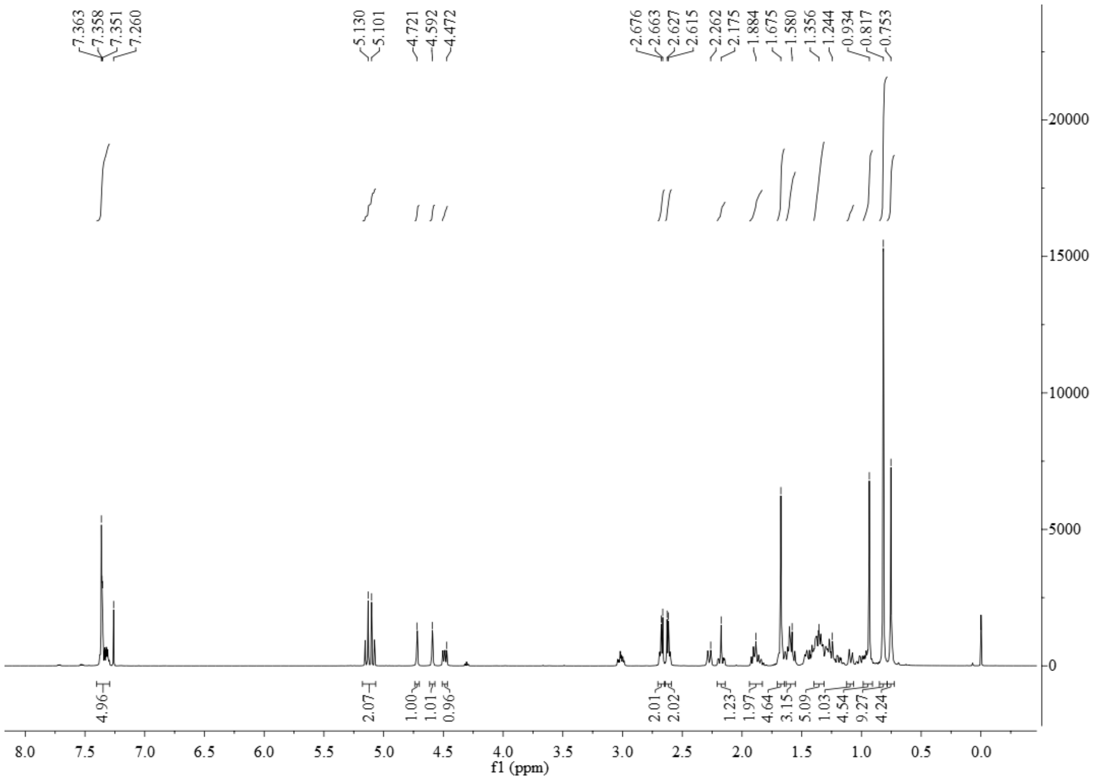

^{13}C-NMR spectra of Compound **3**

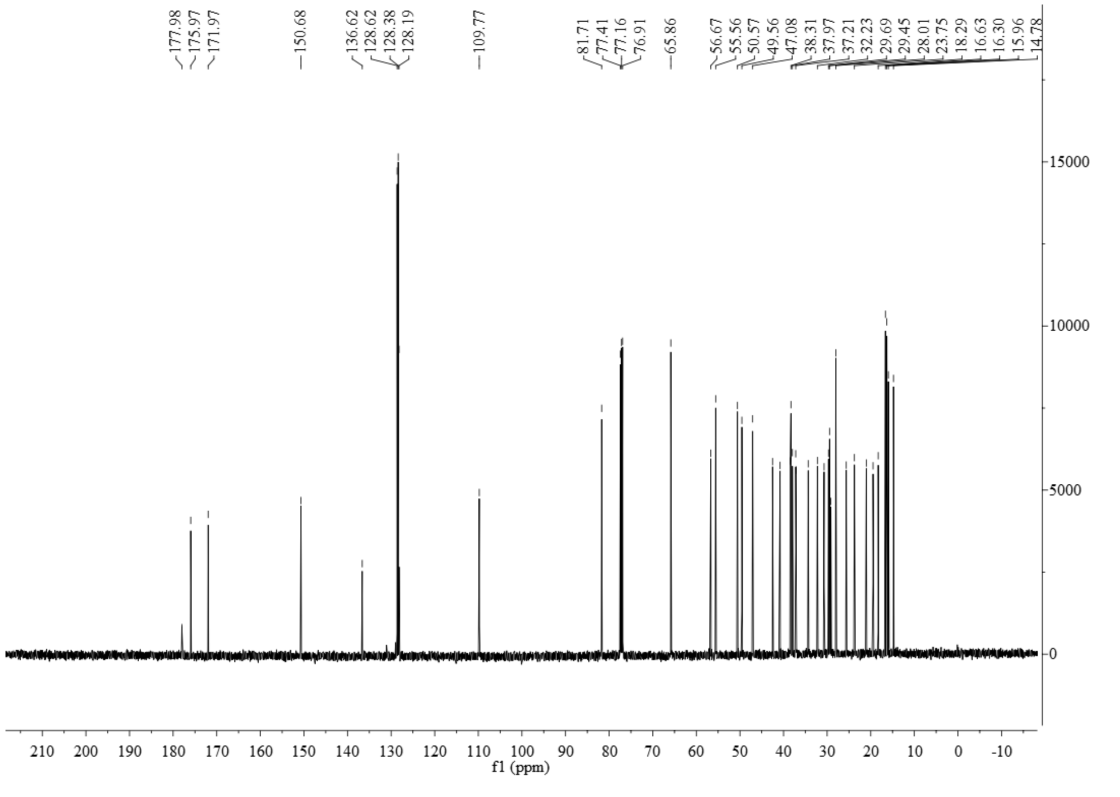

Compound 4a

^{1}H-NMR spectra of Compound **4a**

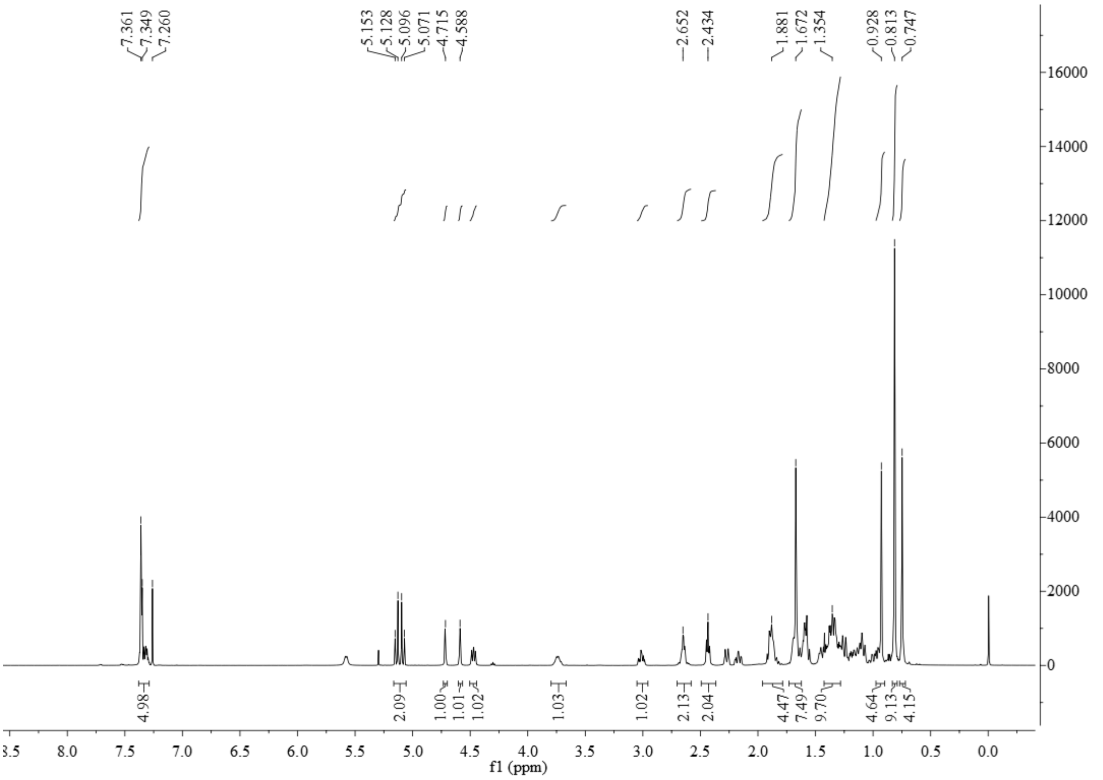

^{13}C-NMR spectra of Compound **4a**

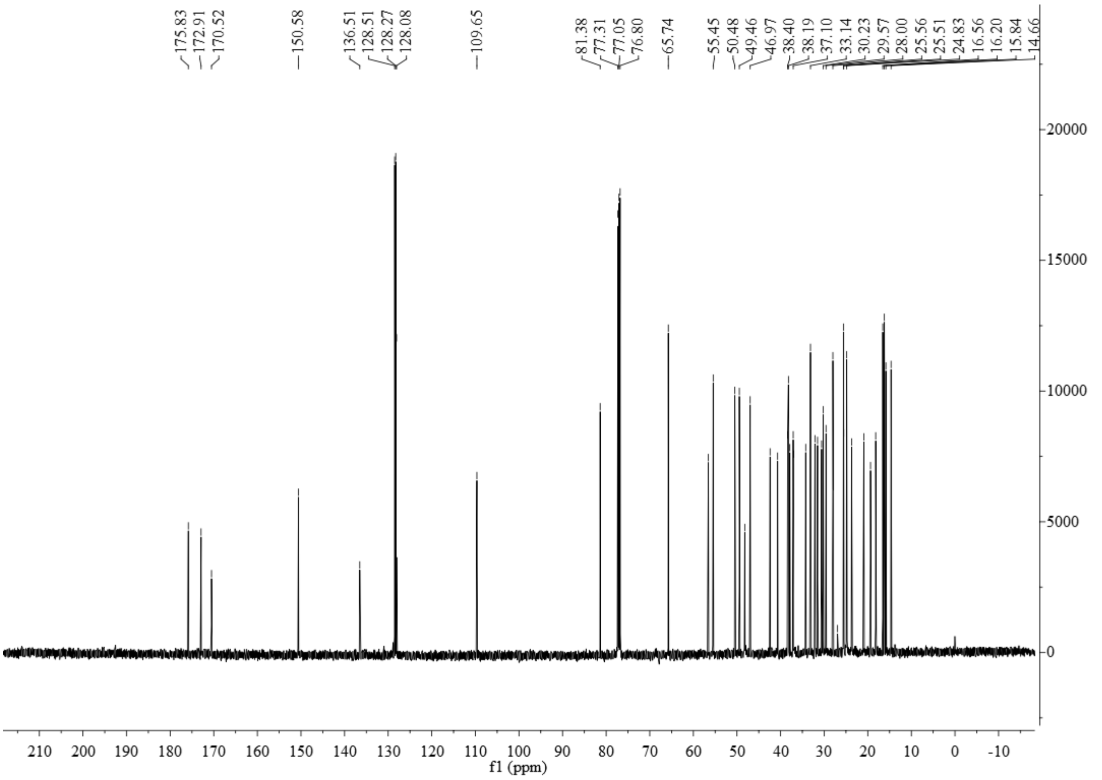

Compound 4b

^{1}H-NMR spectra of Compound **4b**

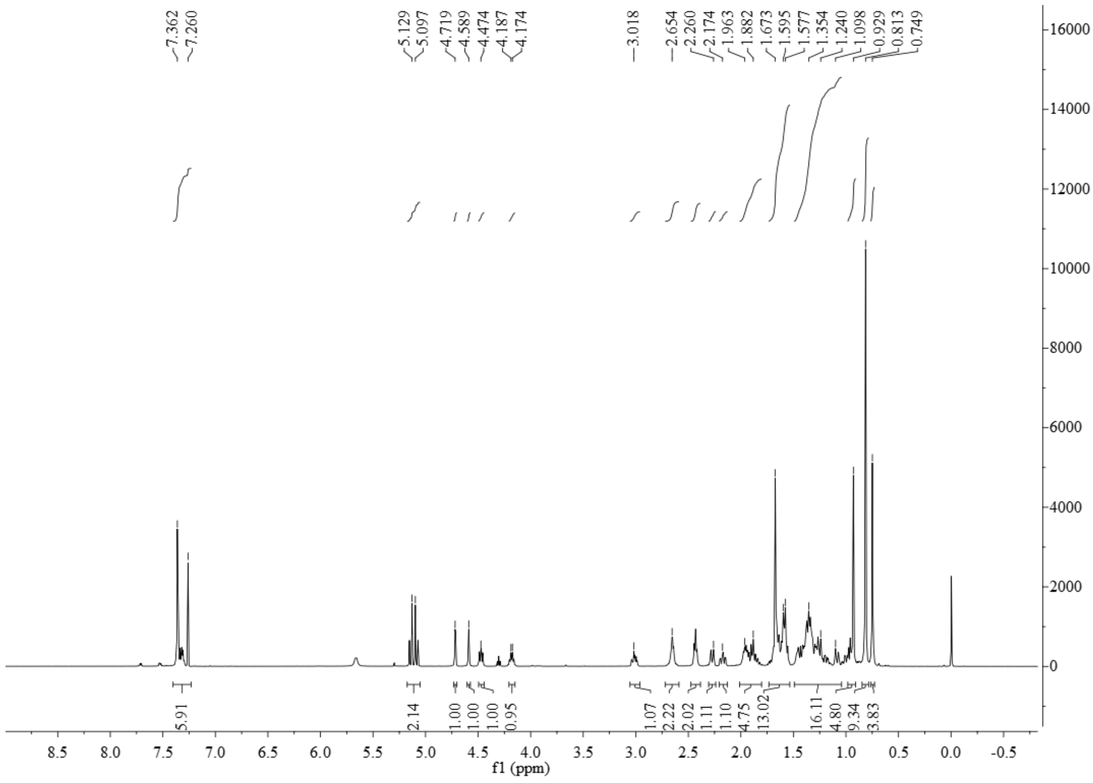

^{13}C-NMR spectra of Compound **4b**

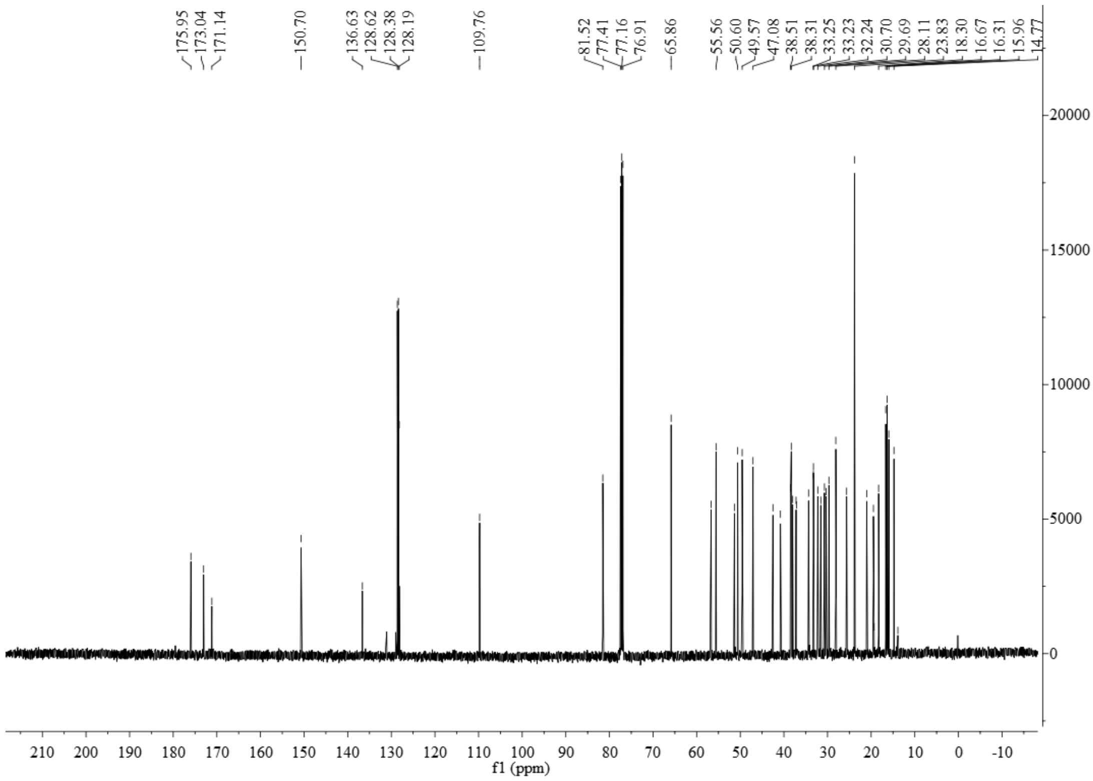

Compound 4c

^{1}H-NMR spectra of Compound **4c**

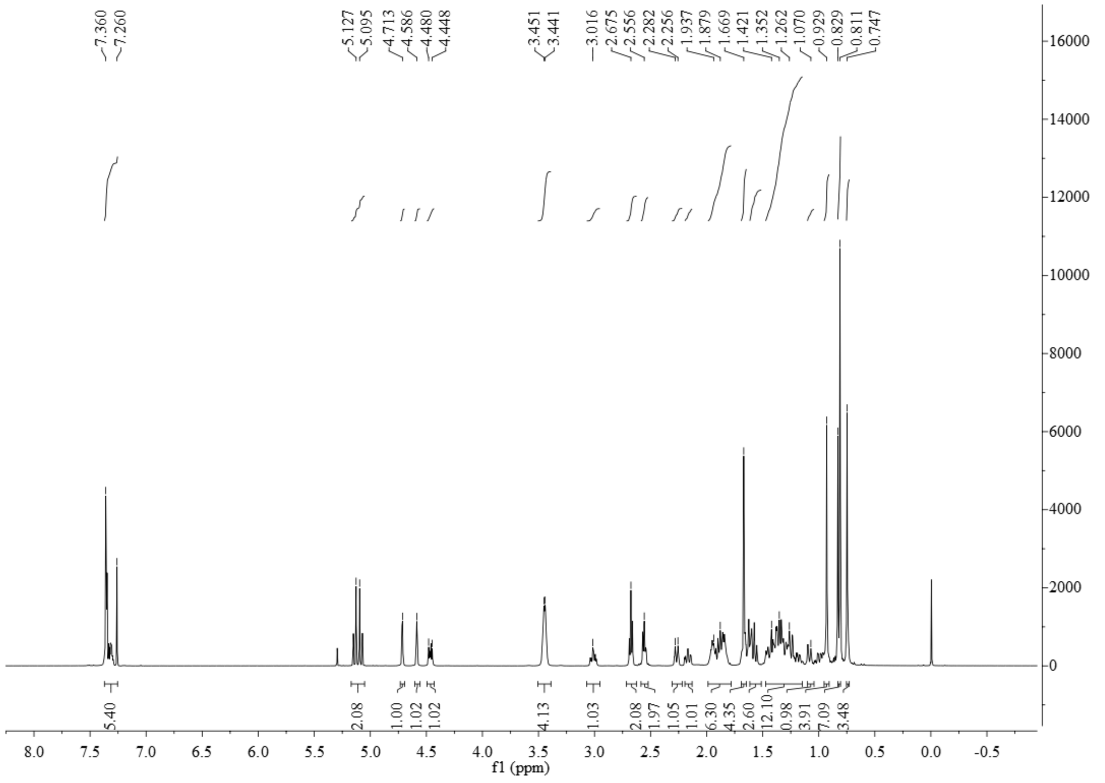

^{13}C-NMR spectra of Compound **4c**

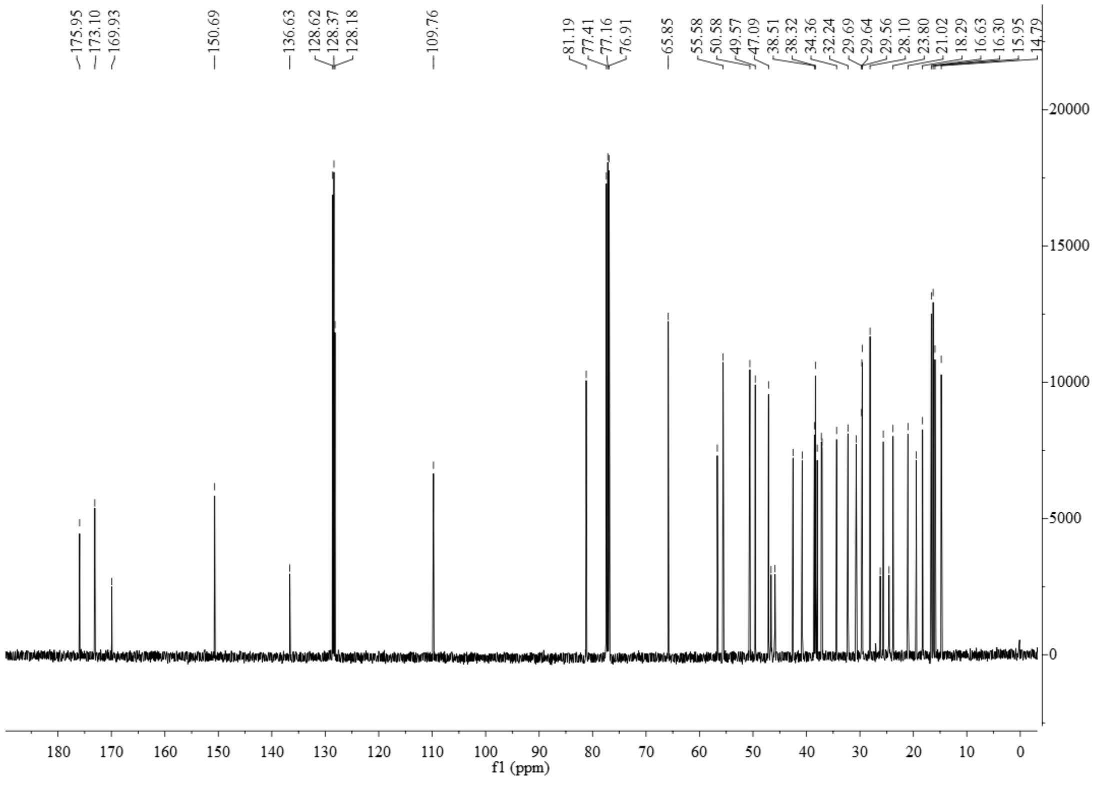

Compound 4d

^{1}H-NMR spectra of Compound **4d**

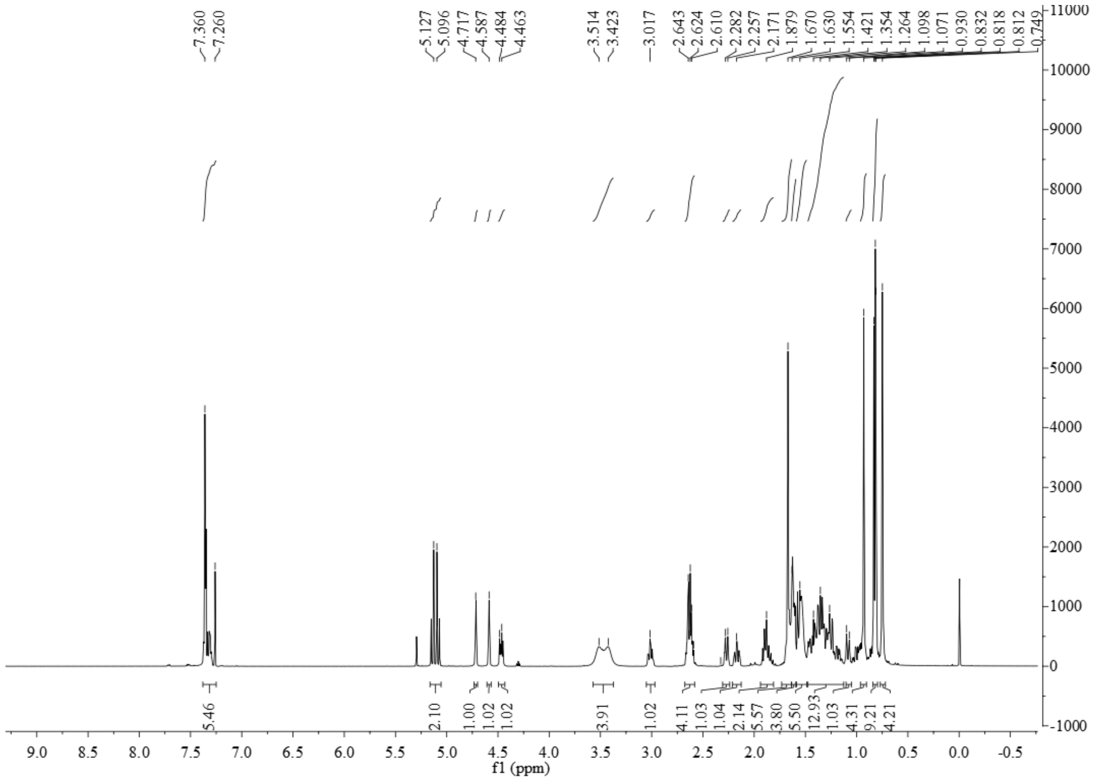

^{13}C-NMR spectra of Compound **4d**

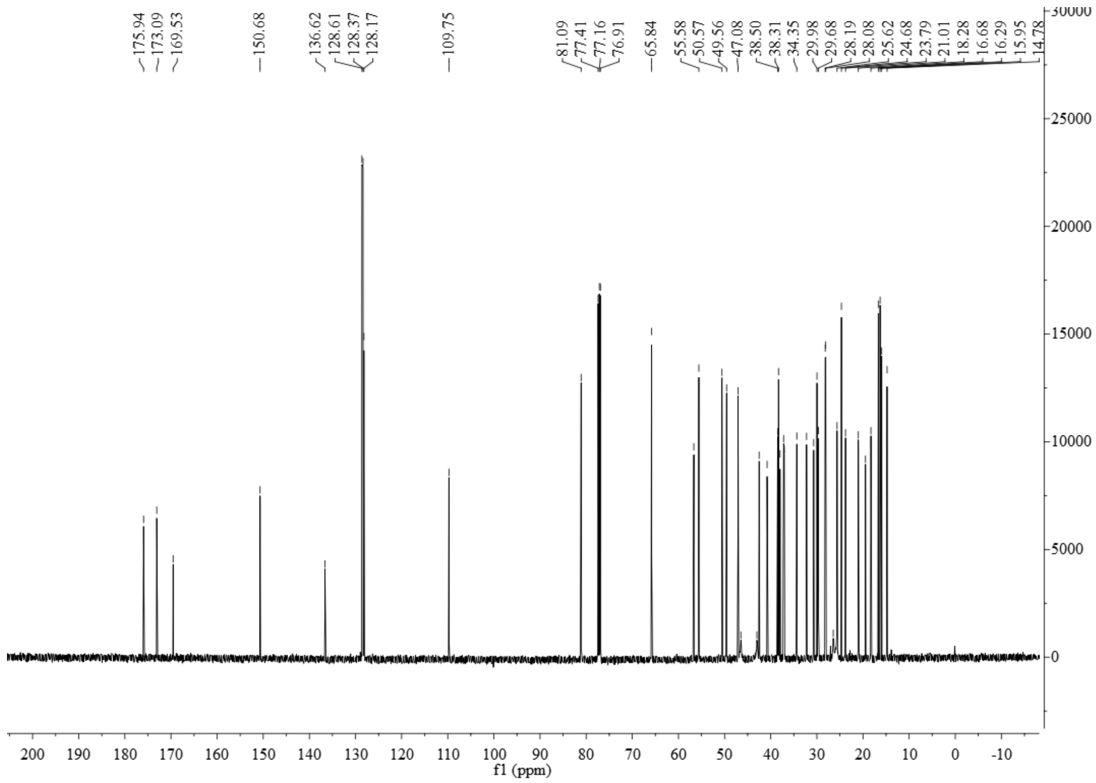

Compound 5a

^{1}H-NMR spectra of Compound **5a**

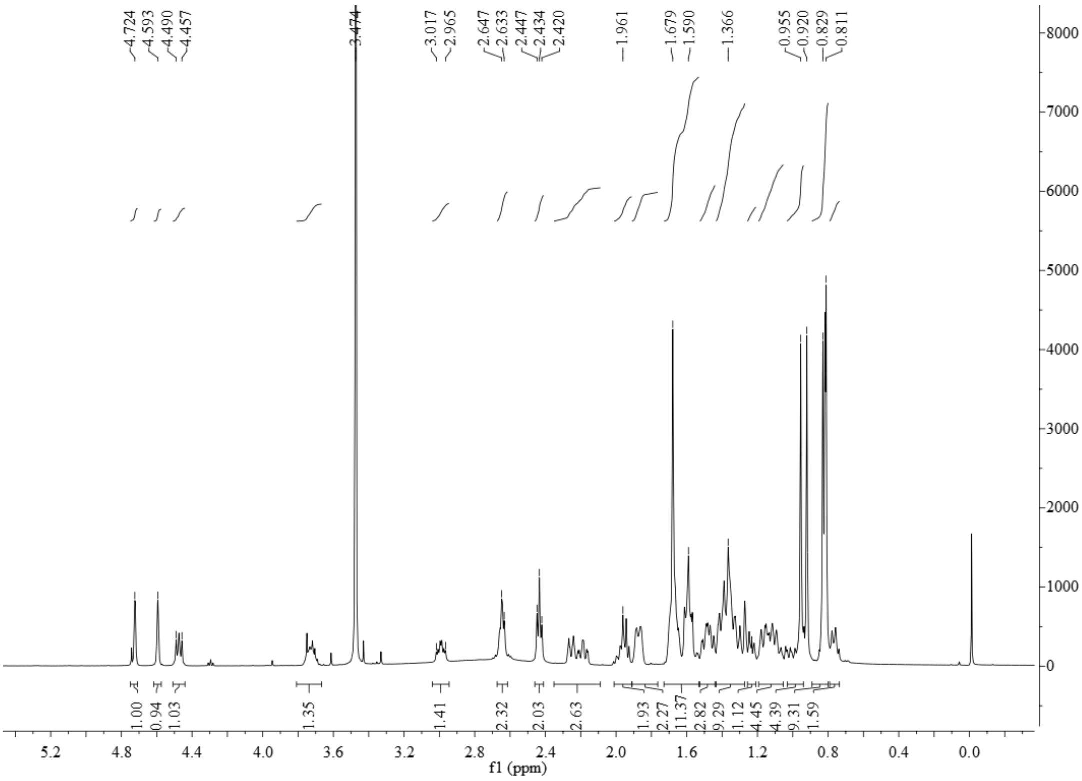

^{13}C-NMR spectra of Compound **5a**

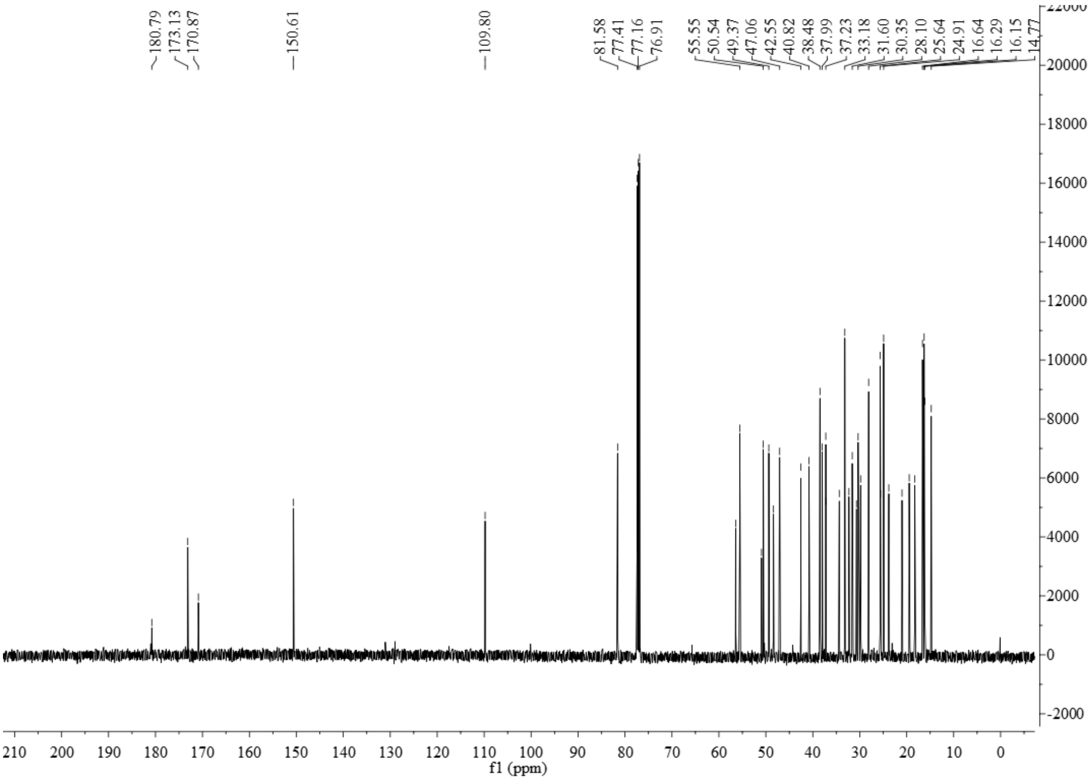

Compound 5b

^{1}H-NMR spectra of Compound **5b**

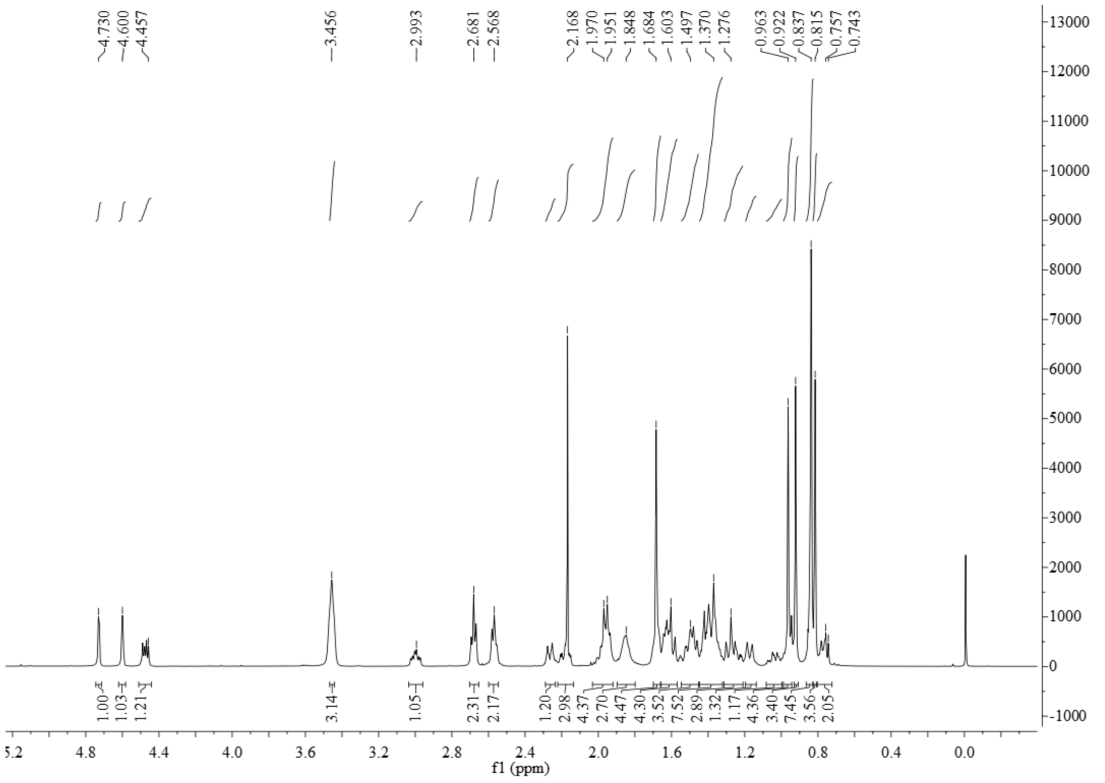

^{13}C-NMR spectra of Compound **5b**

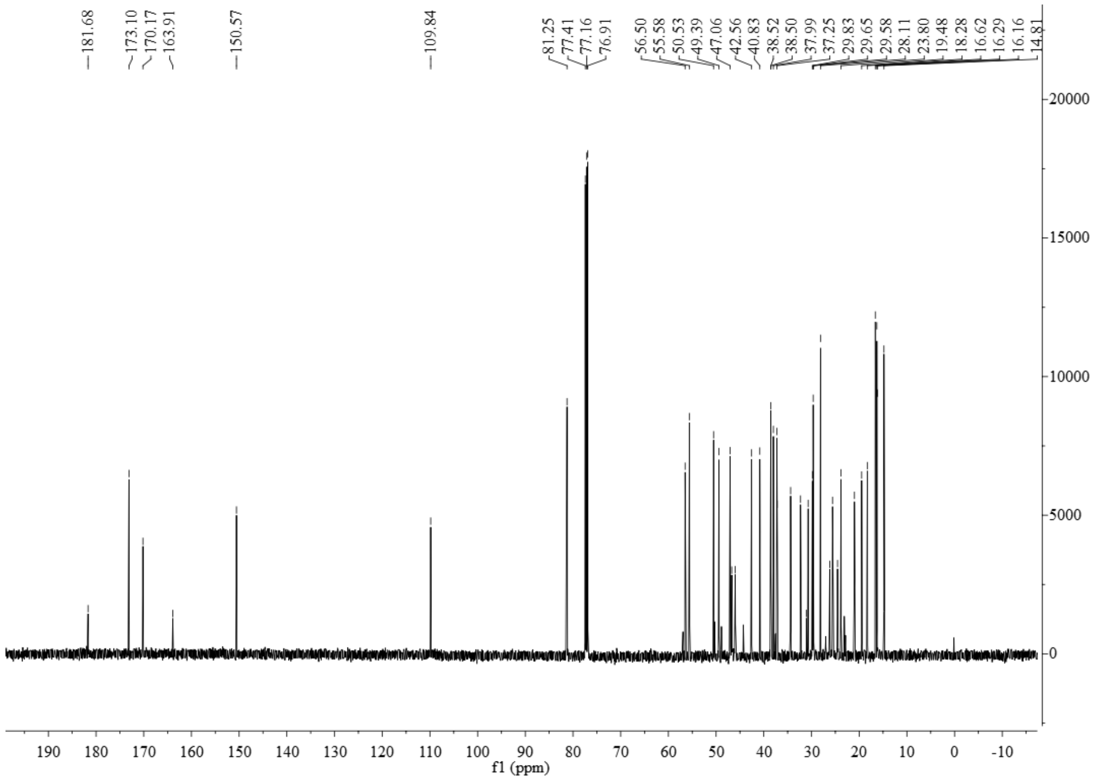

Compound 5c

^{1}H-NMR spectra of Compound **5c**

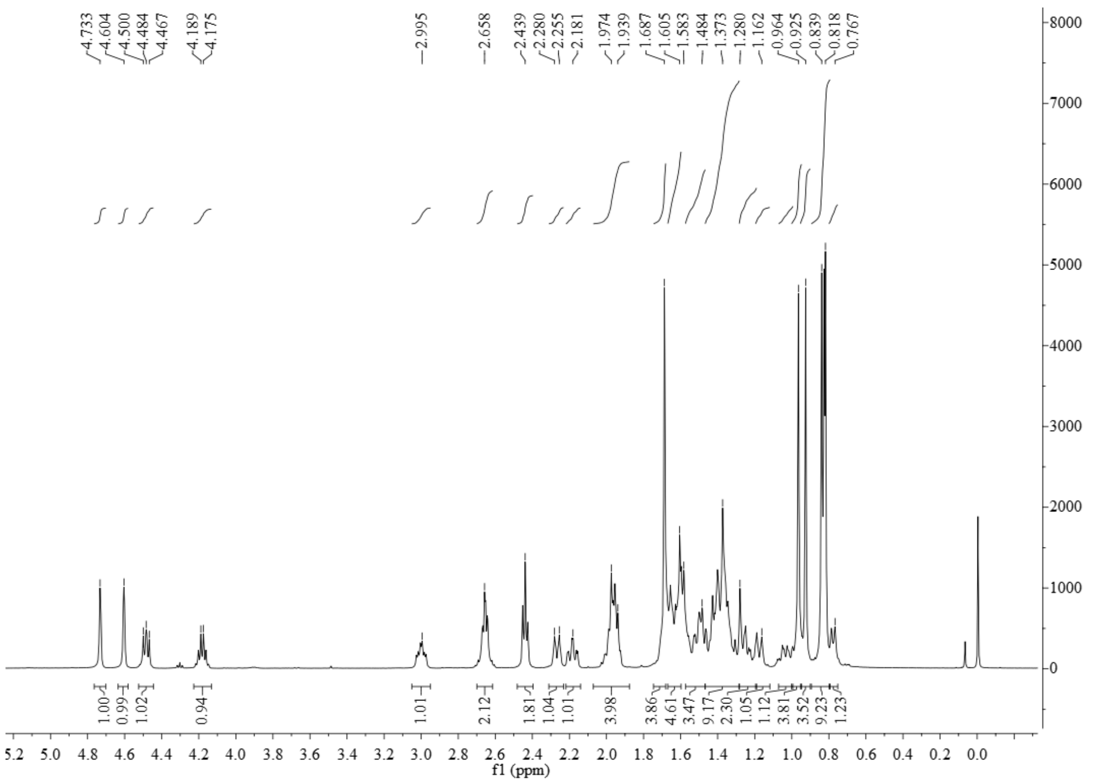

^{13}C-NMR spectra of Compound **5c**

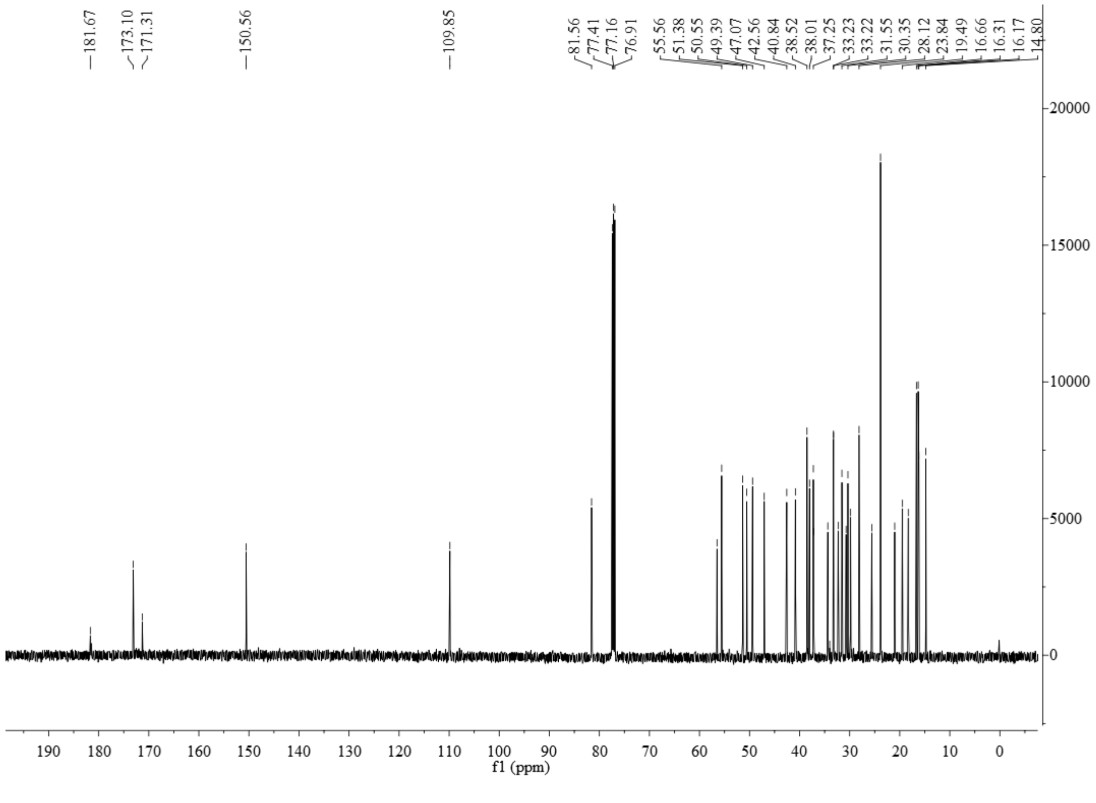

Compound 5d

^{1}H-NMR spectra of Compound **5d**

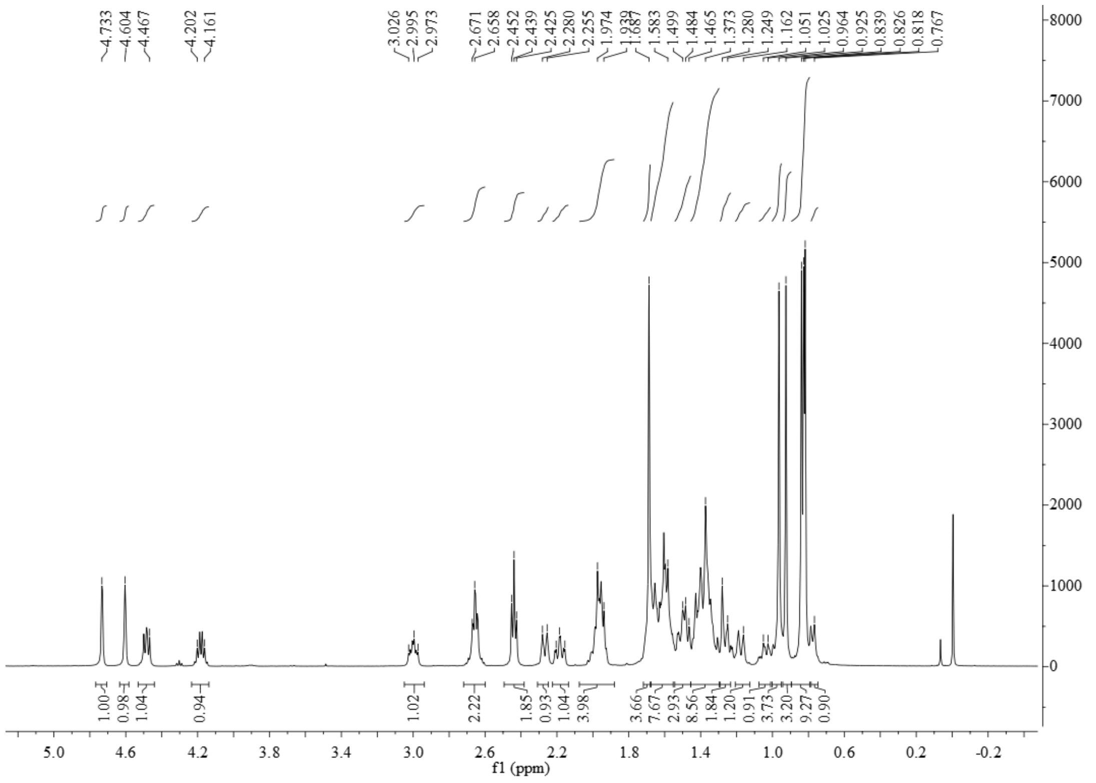

^{13}C-NMR spectra of Compound **5d**

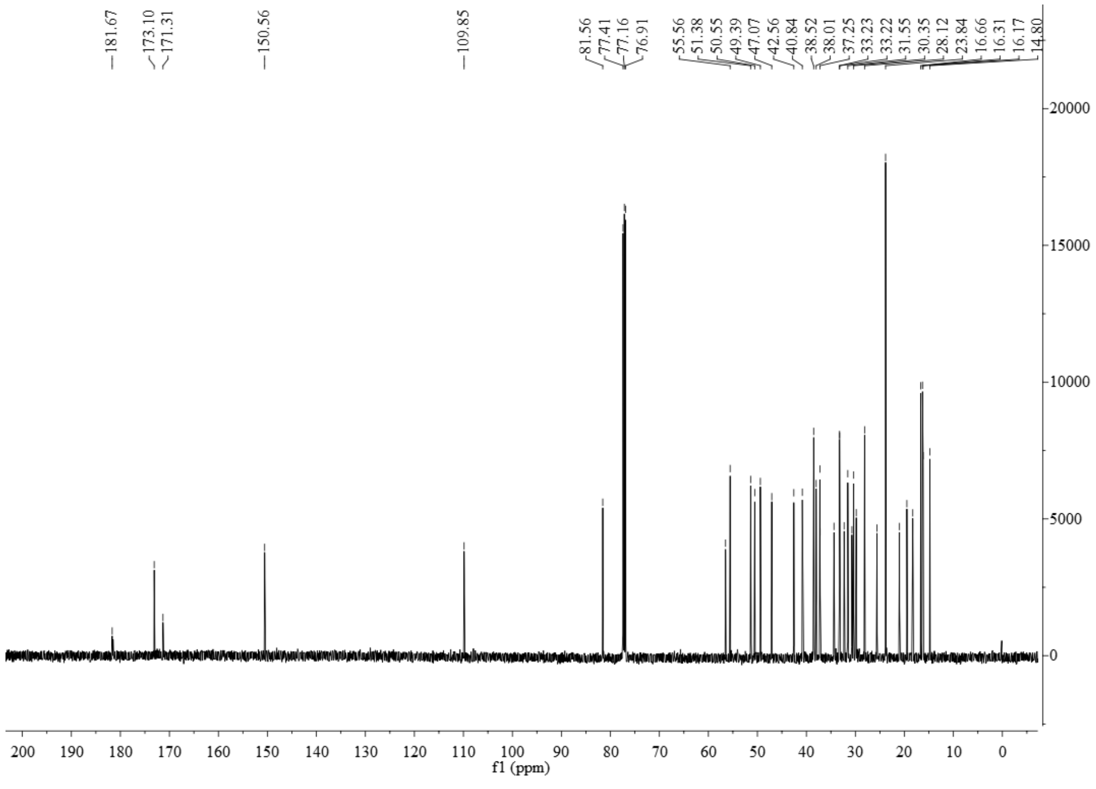

Compound 6

^{1}H-NMR spectra of Compound **6**

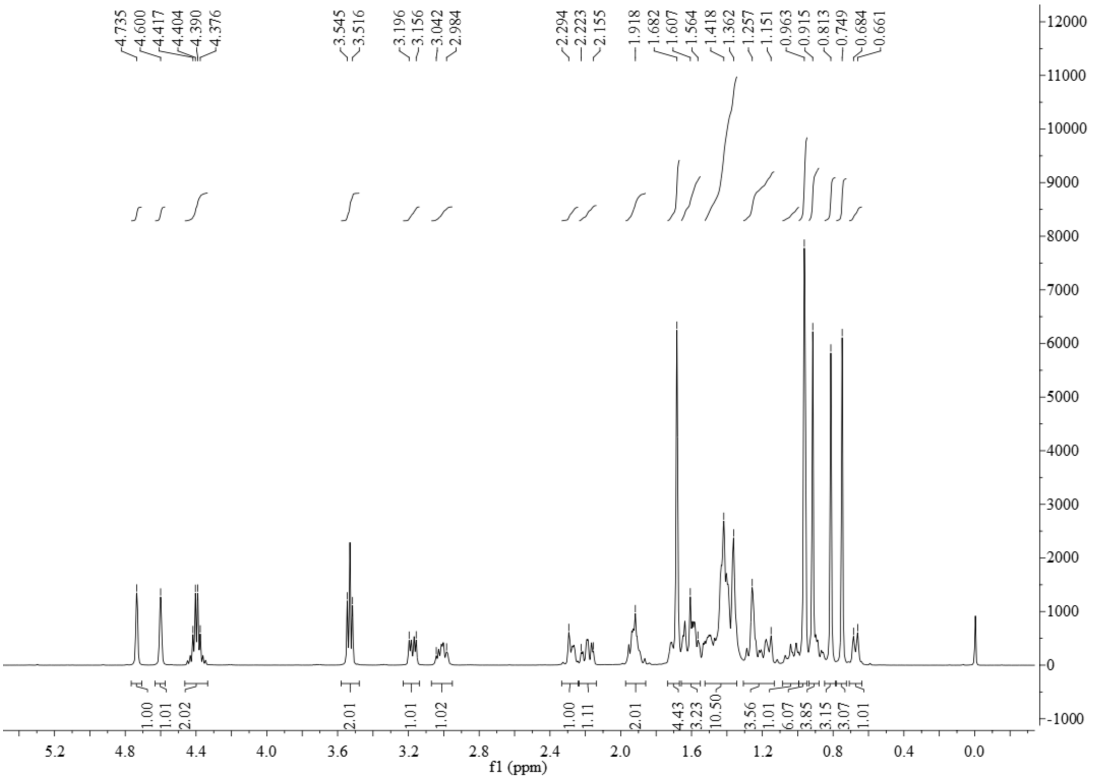

^{13}C-NMR spectra of Compound **6**

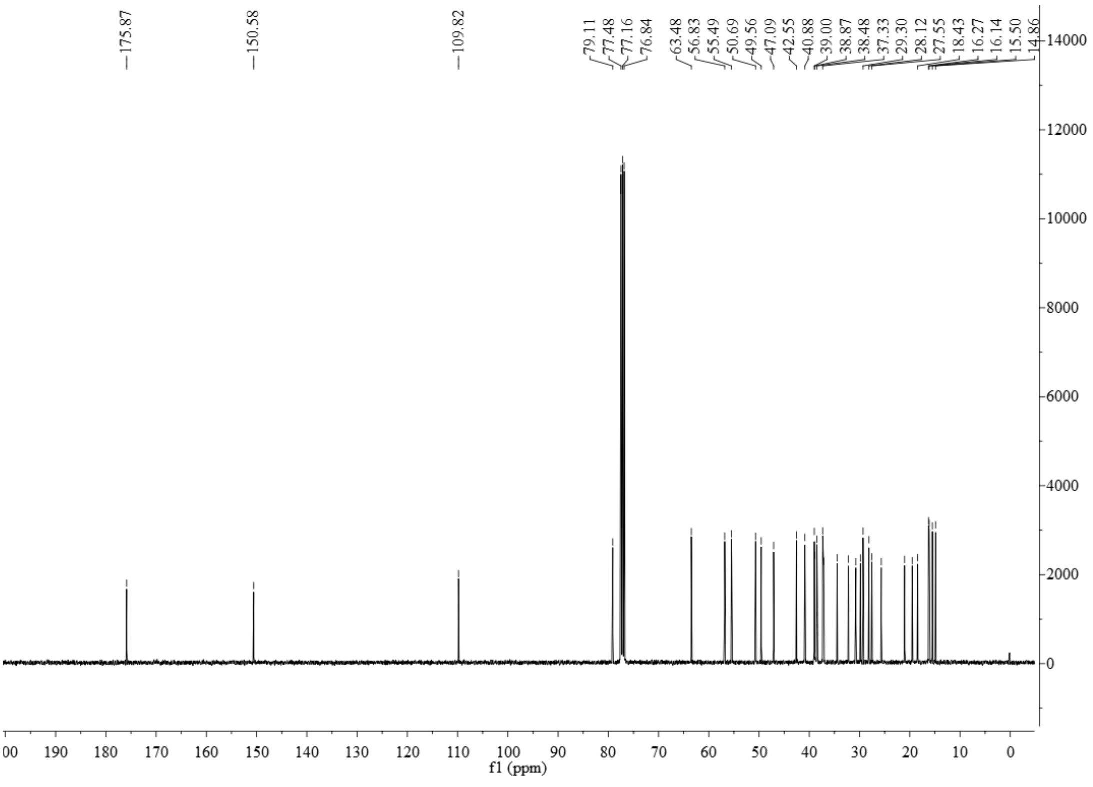

Compound 7a

^{1}H-NMR spectra of Compound **7a**

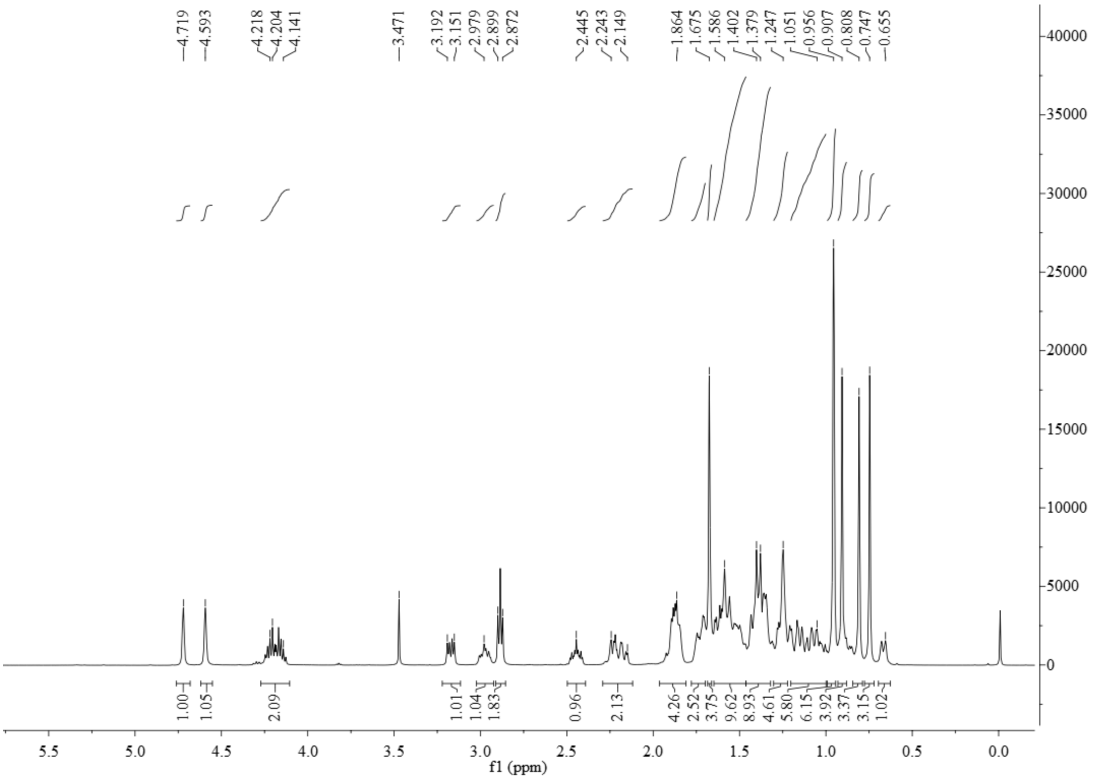

^{13}C-NMR spectra of Compound **7a**

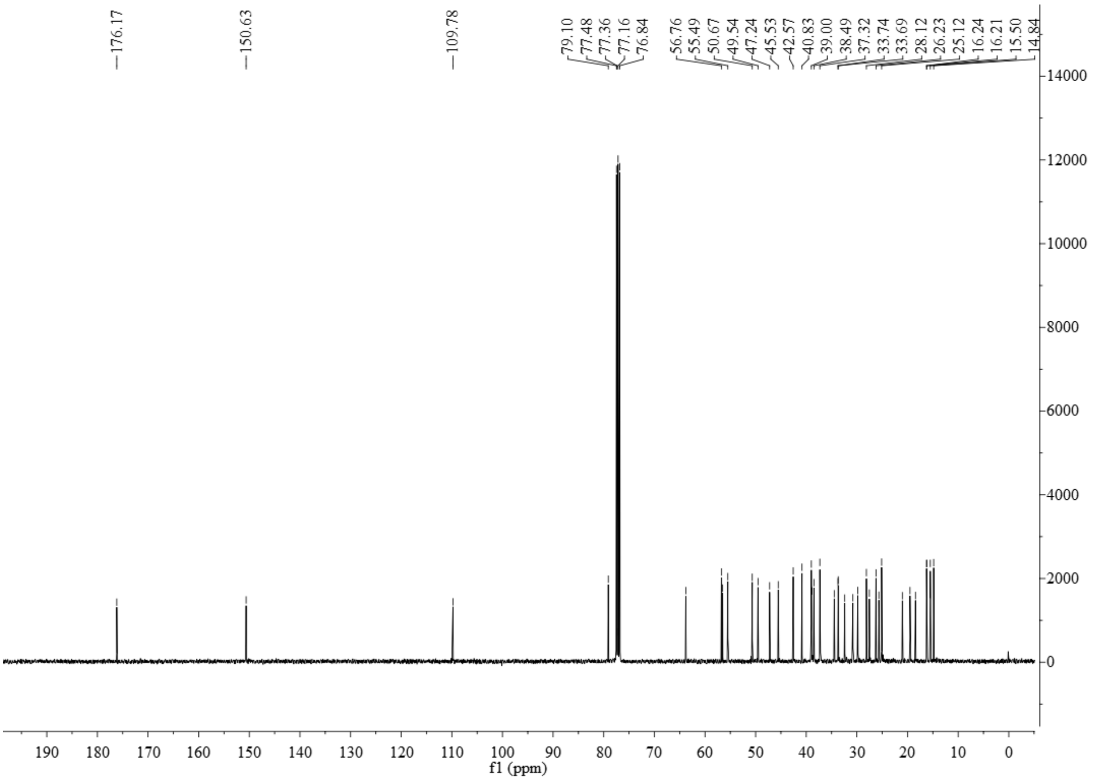

Compound 7c

^{1}H-NMR spectra of Compound **7c**

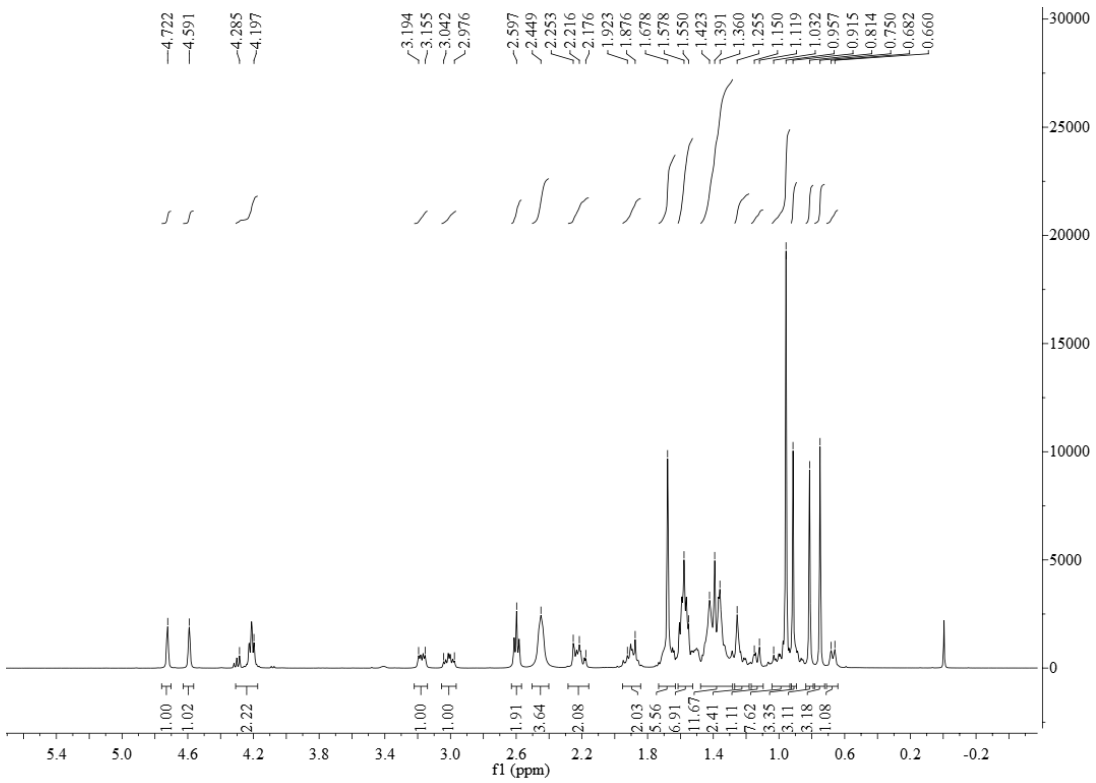

^{13}C-NMR spectra of Compound **7c**

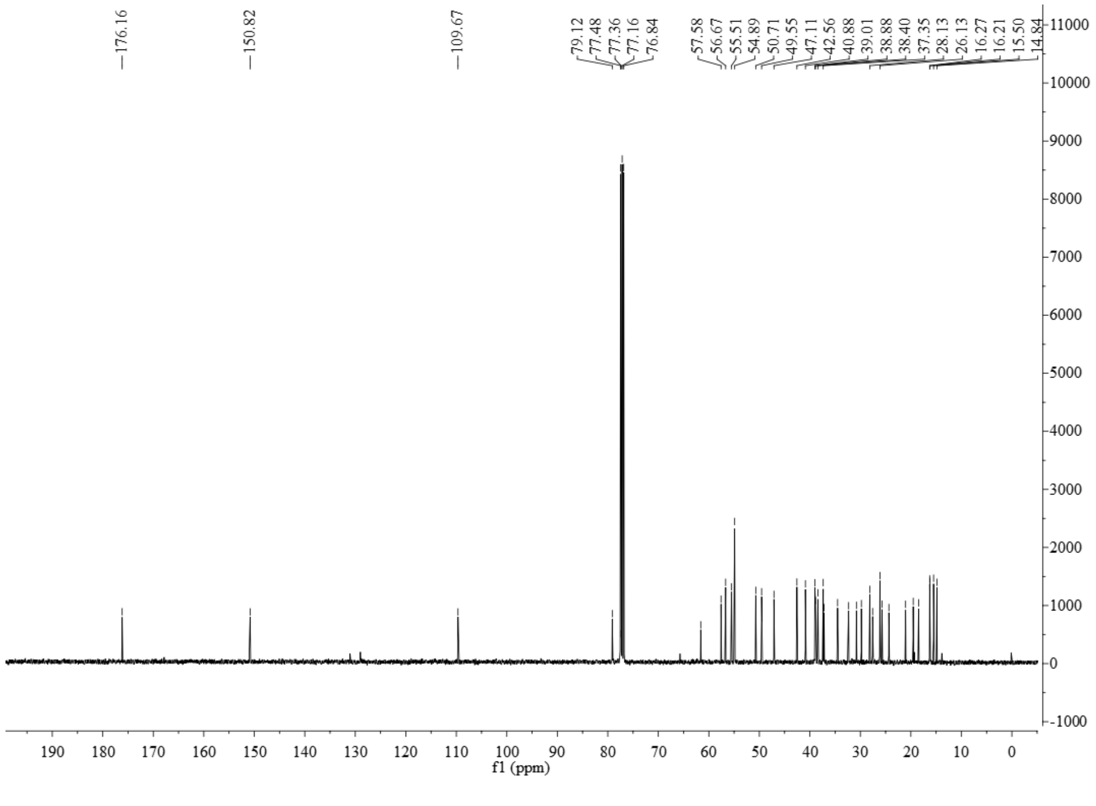

Compound 7d

^{1}H-NMR spectra of Compound **7d**

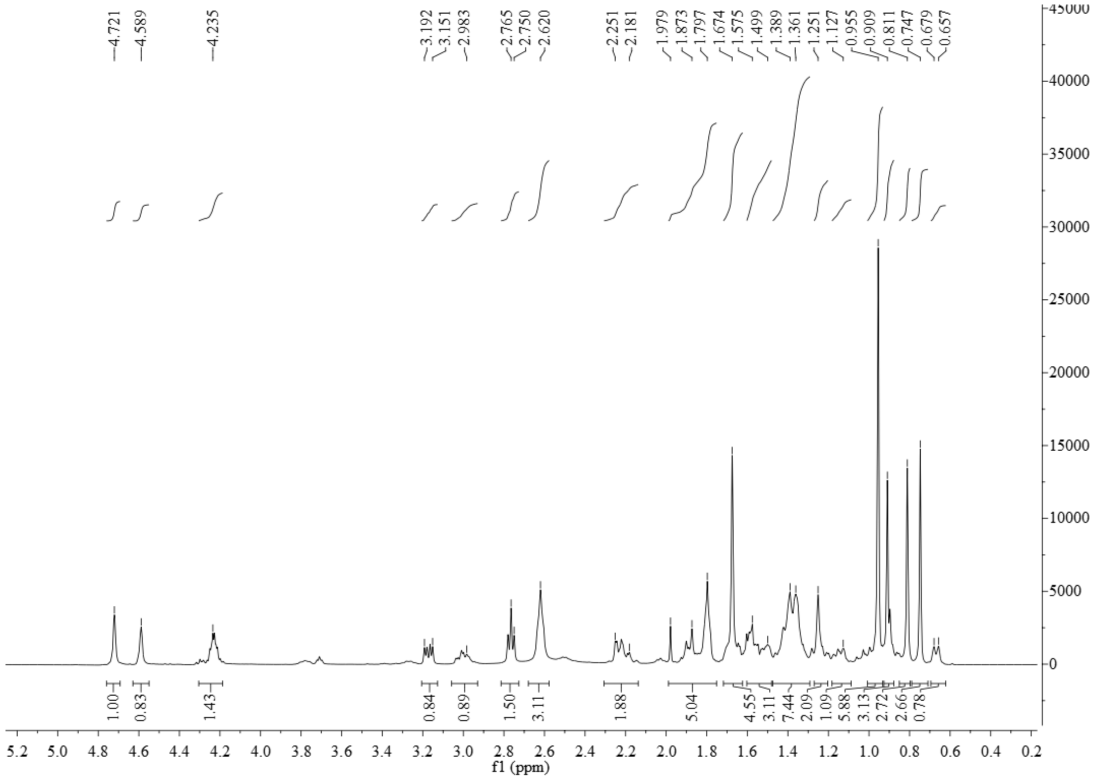

^{13}C-NMR spectra of Compound **7d**

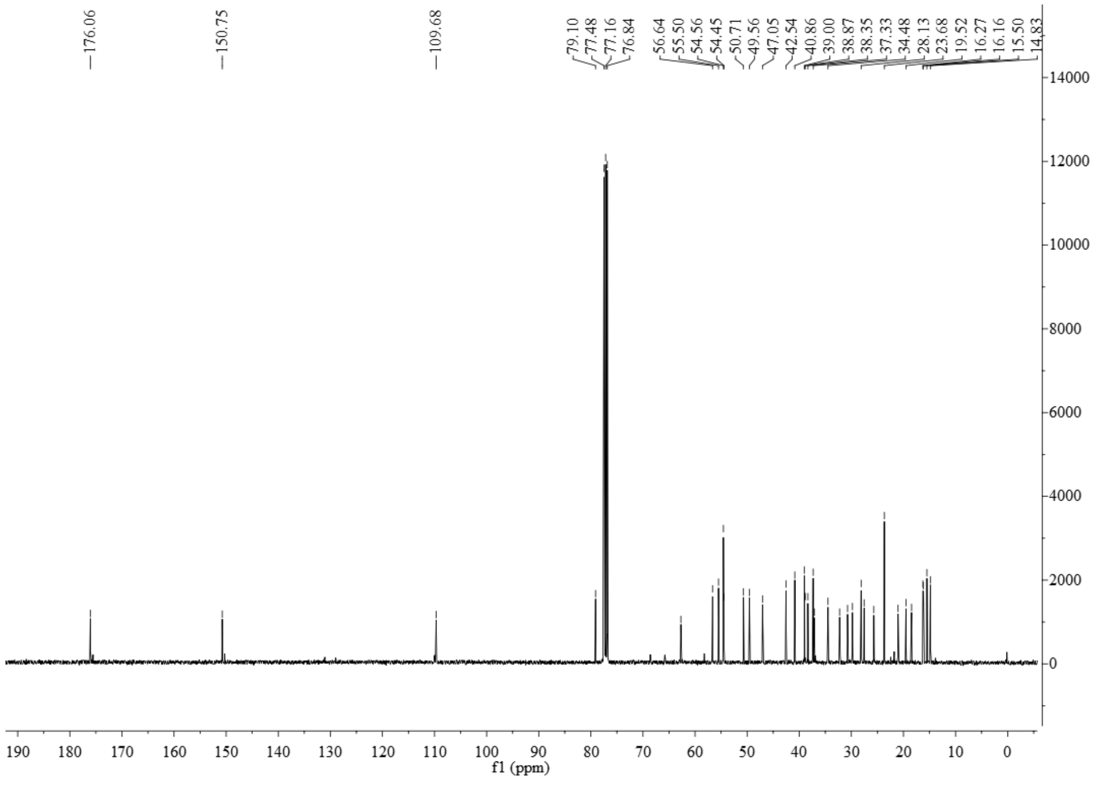

Compound 7e

^{1}H-NMR spectra of Compound **7e**

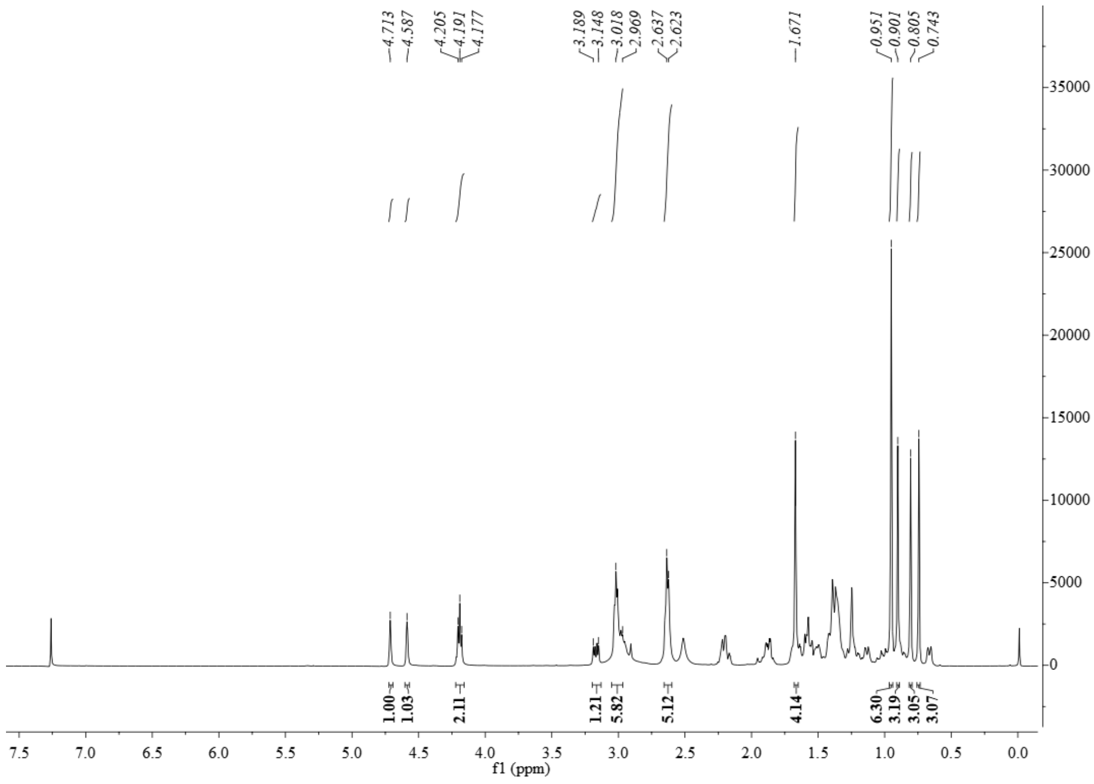

^{13}C-NMR spectra of Compound **7e**

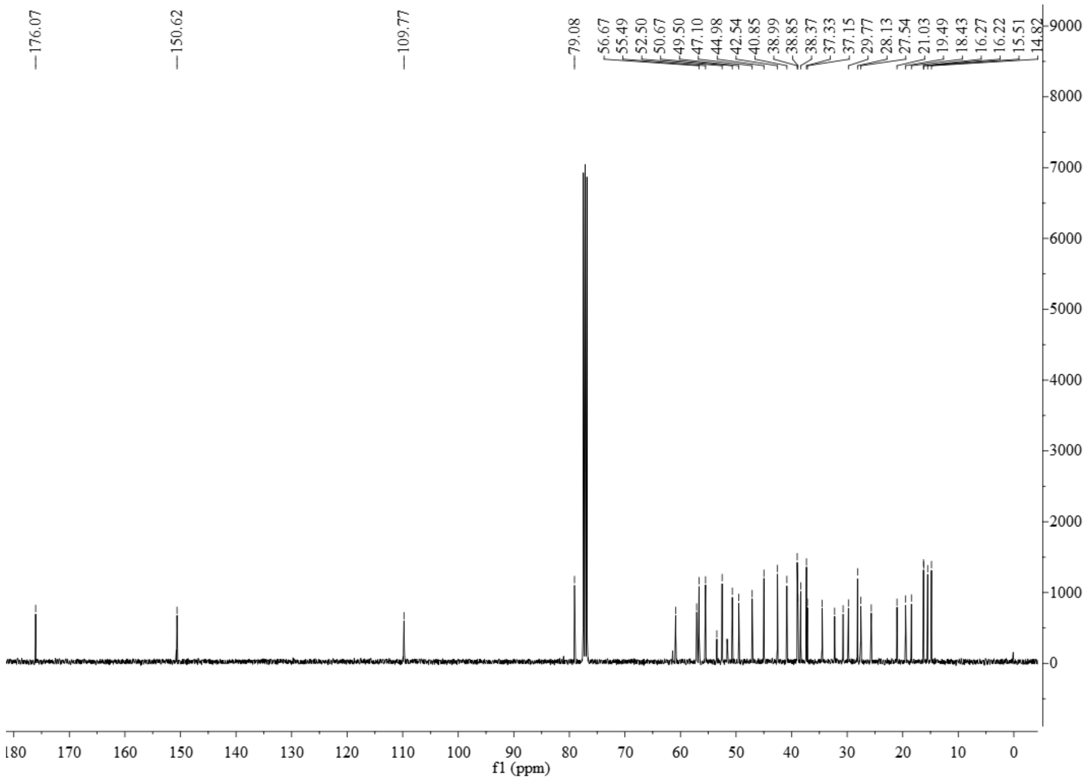

Compound 8

^{1}H-NMR spectra of Compound **8**

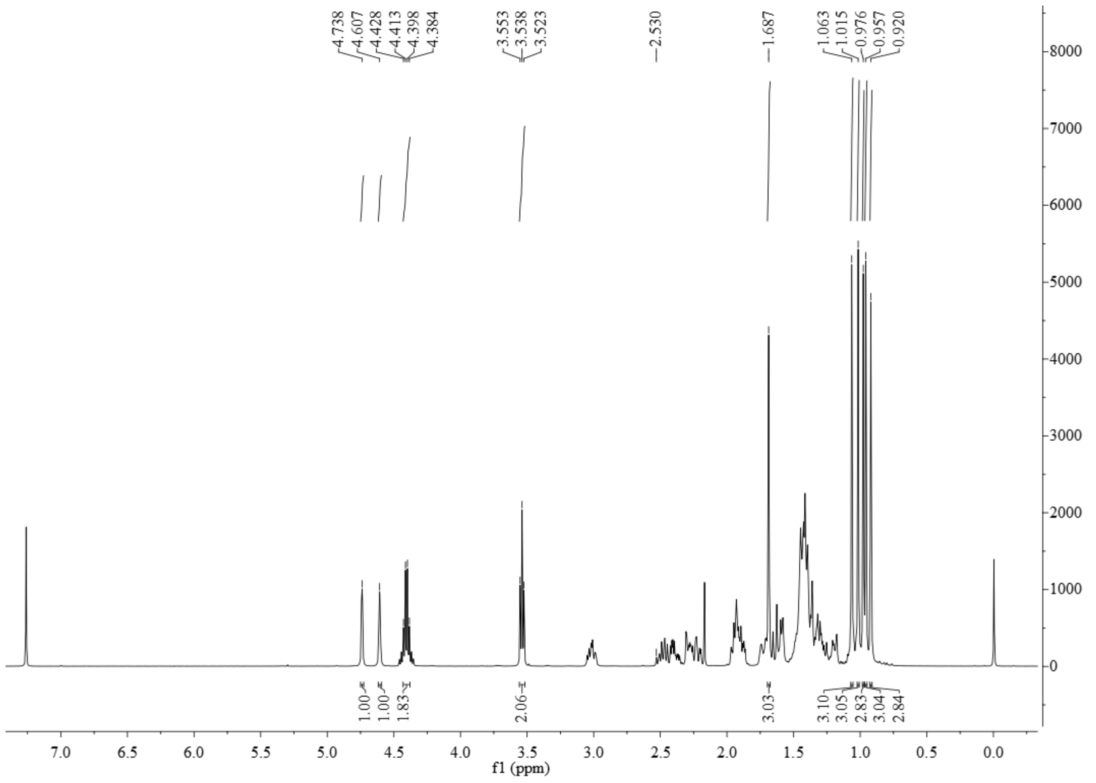

^{13}C-NMR spectra of Compound **8**

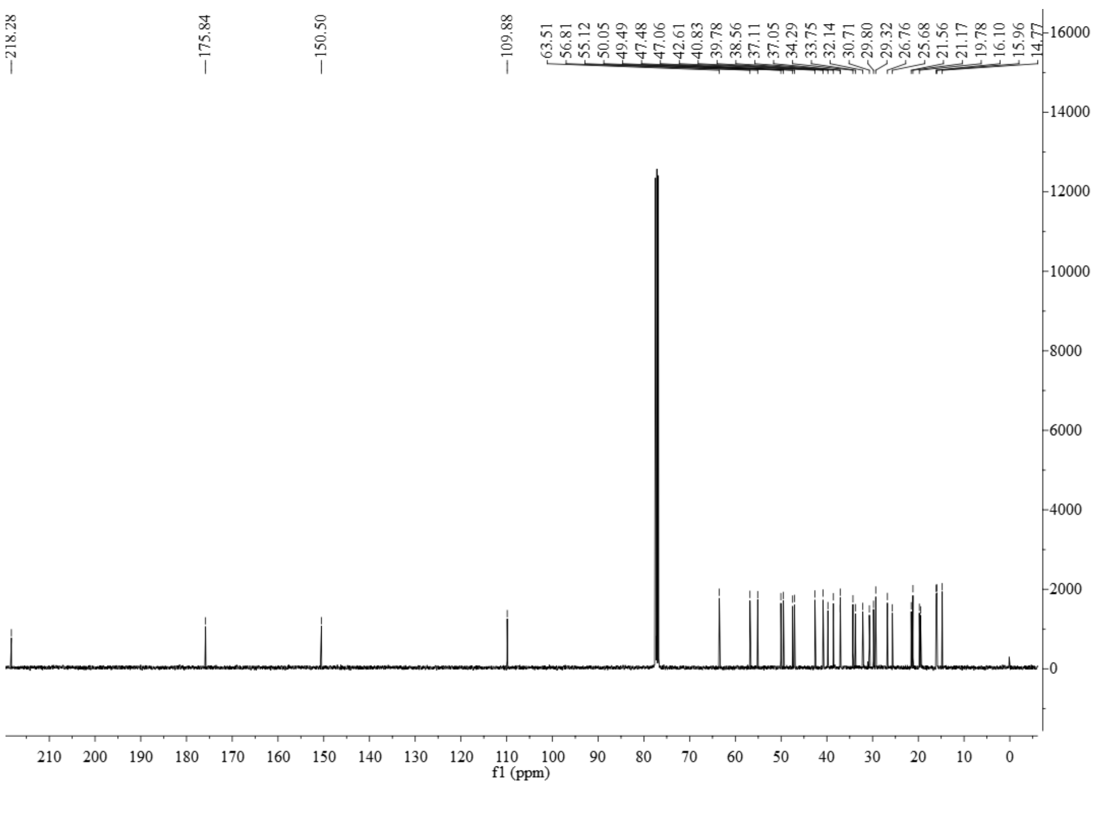

Compound 9a

^{1}H-NMR spectra of Compound **9a**

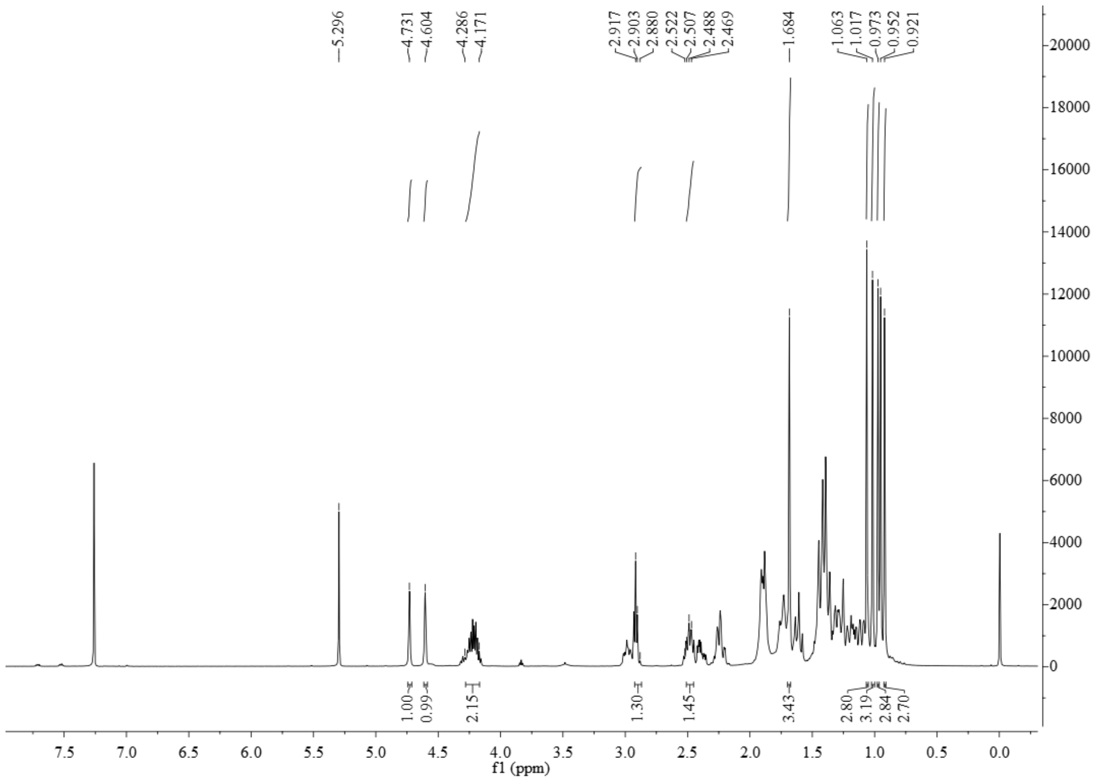

^{13}C-NMR spectra of Compound **9a**

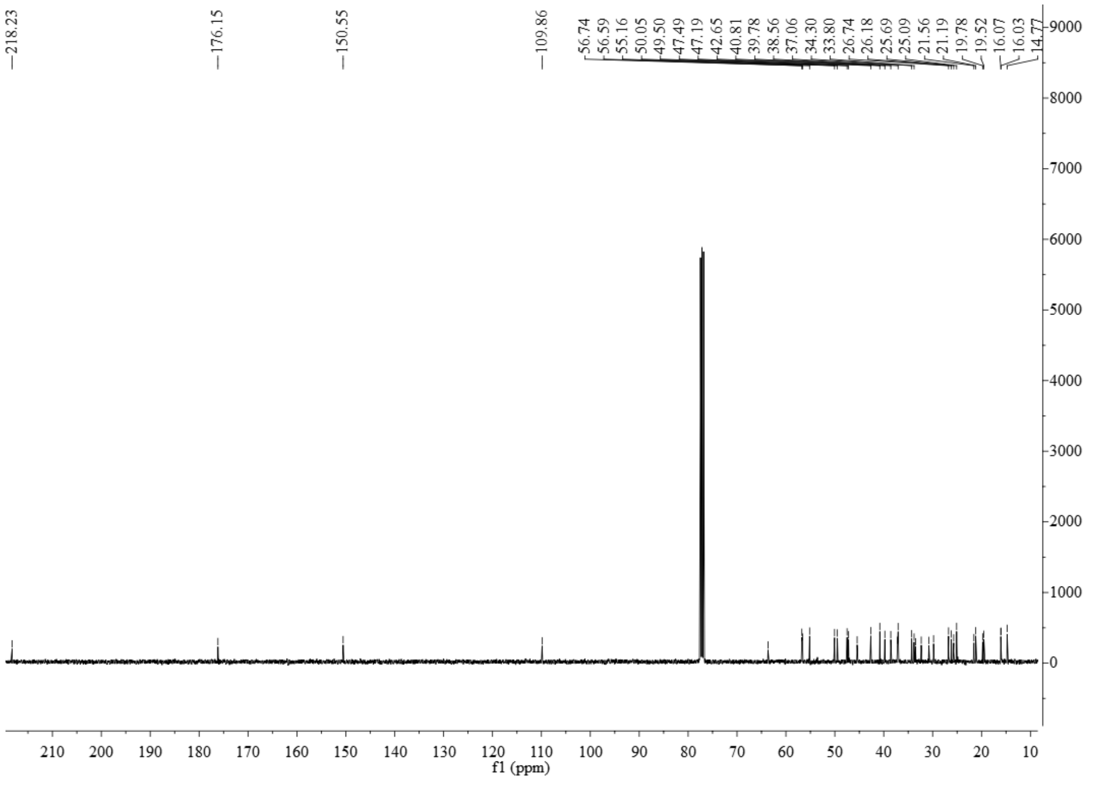

Compound 9b

^{1}H-NMR spectra of Compound **9b**

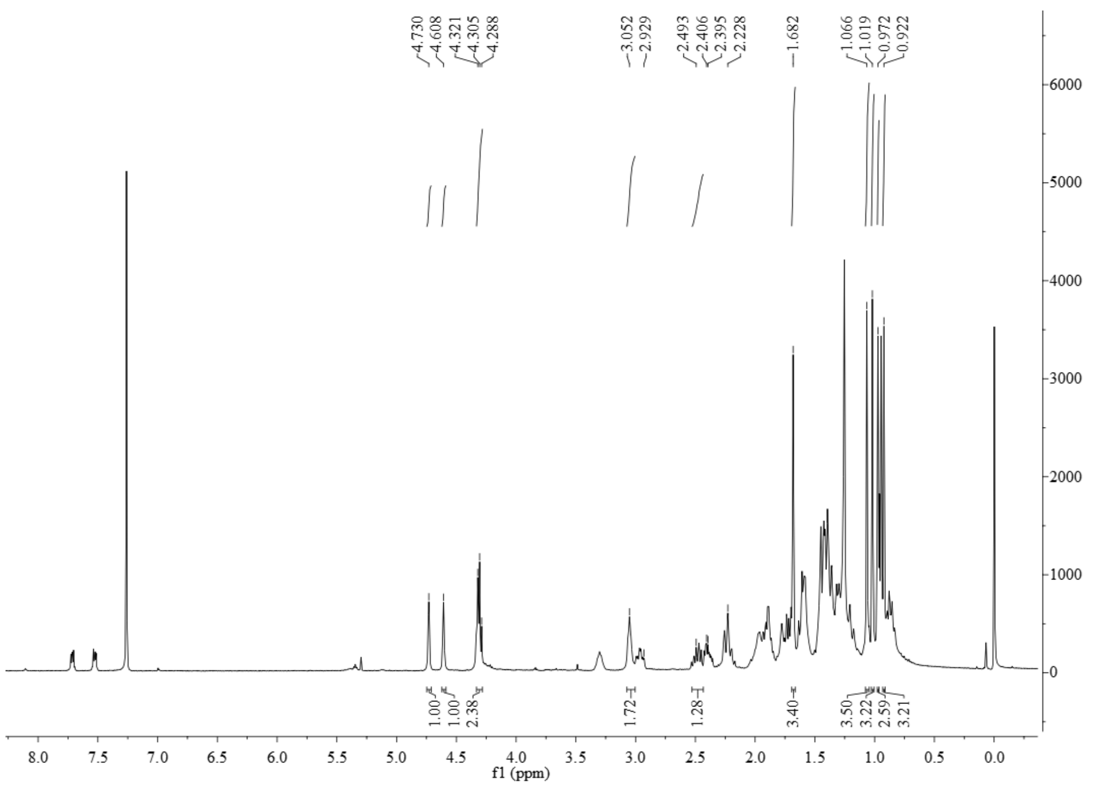

^{13}C-NMR spectra of Compound **9b**

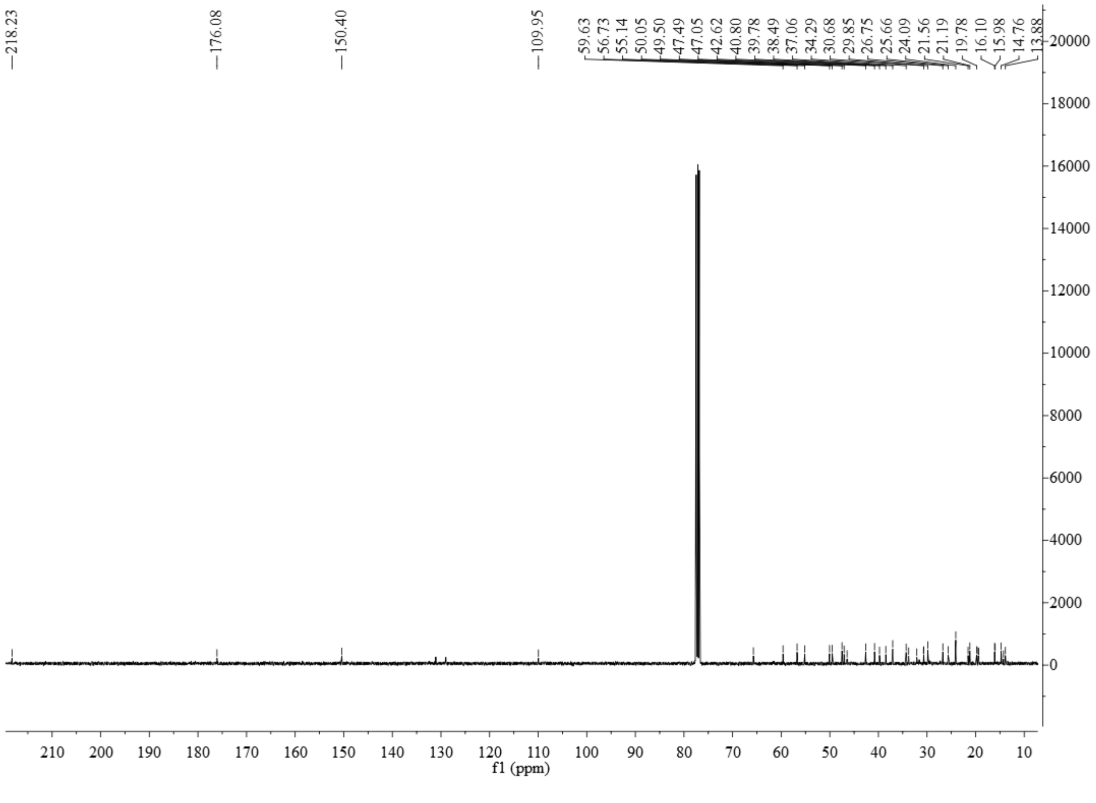

Compound 9c

^{1}H-NMR spectra of Compound **9c**

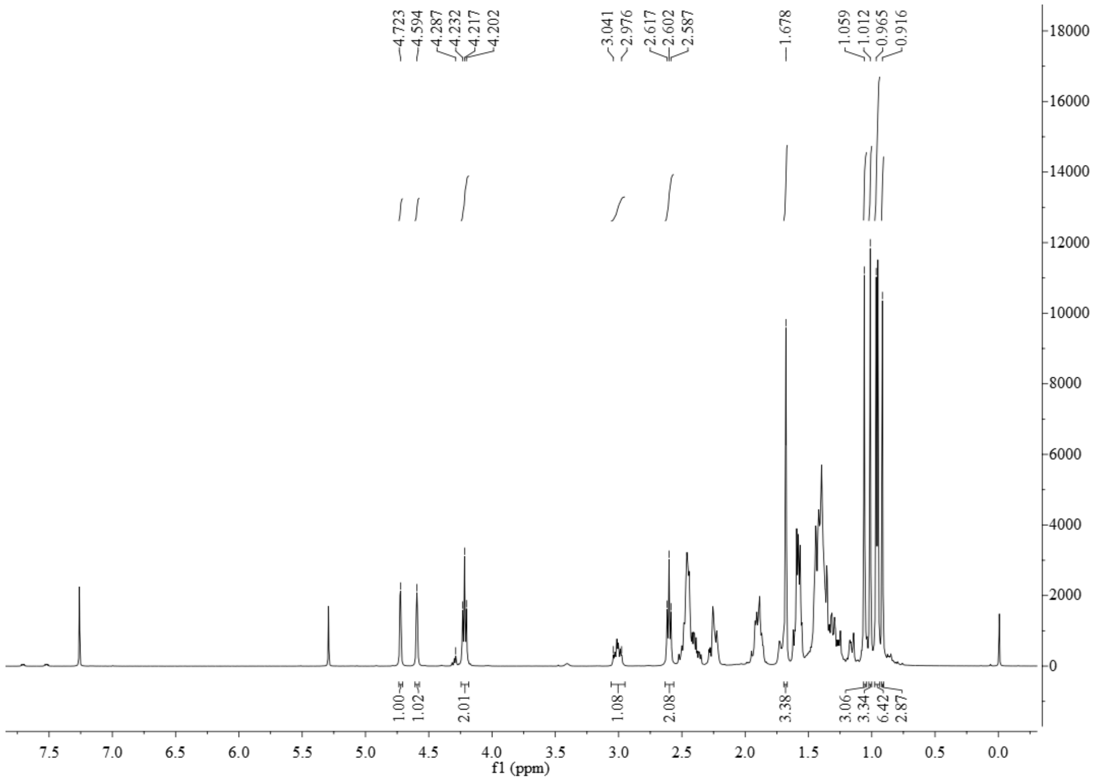

^{13}C-NMR spectra of Compound **9c**

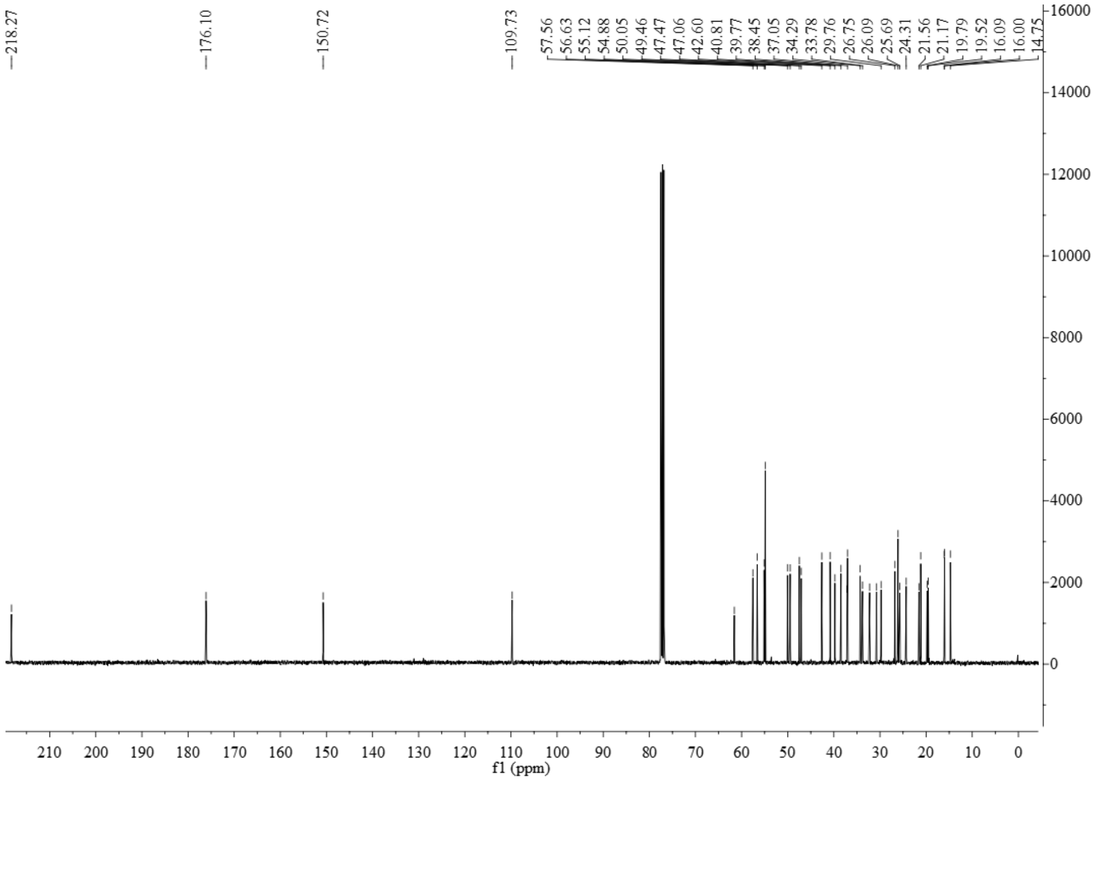

Compound 9d

^{1}H-NMR spectra of Compound **9d**

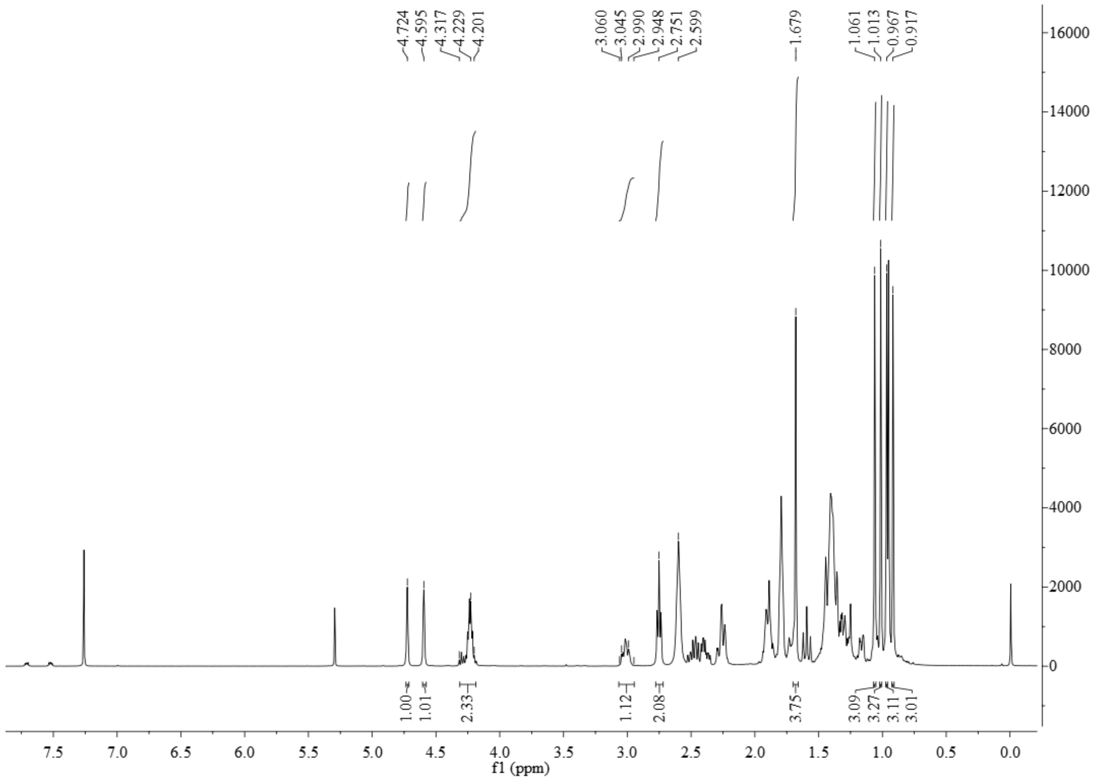

^{13}C-NMR spectra of Compound **9d**

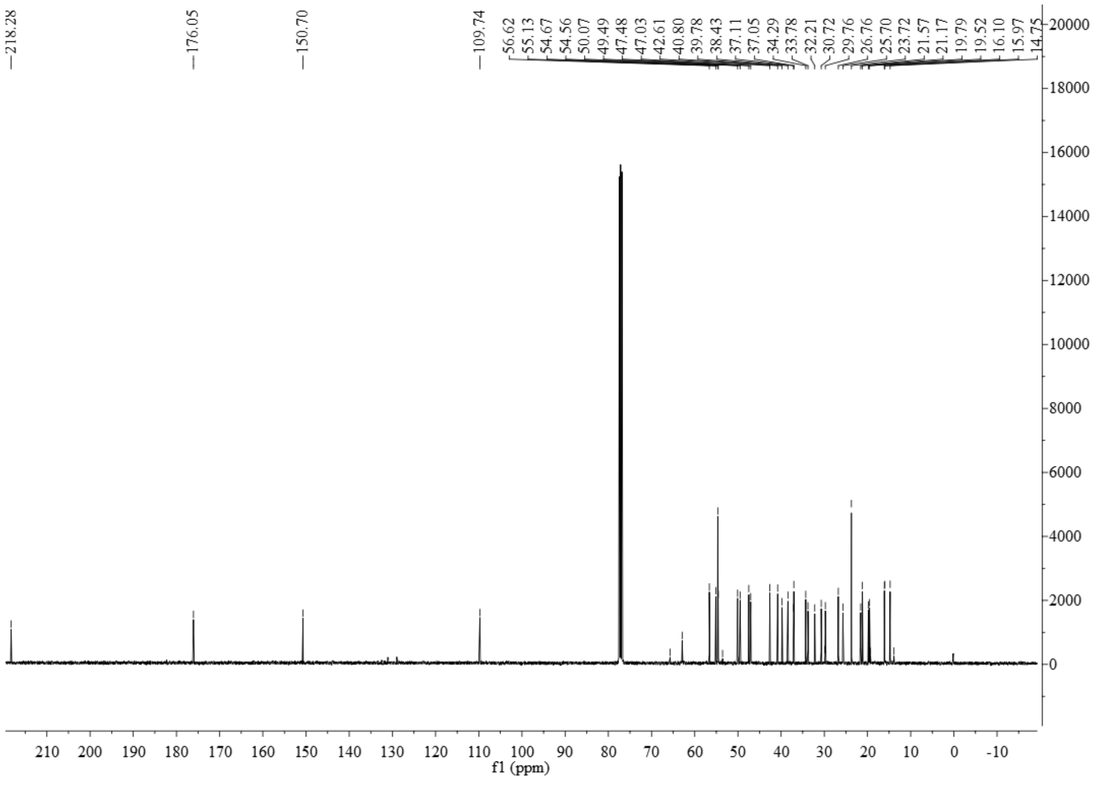

Compound 10

^{1}H-NMR spectra of Compound **10**

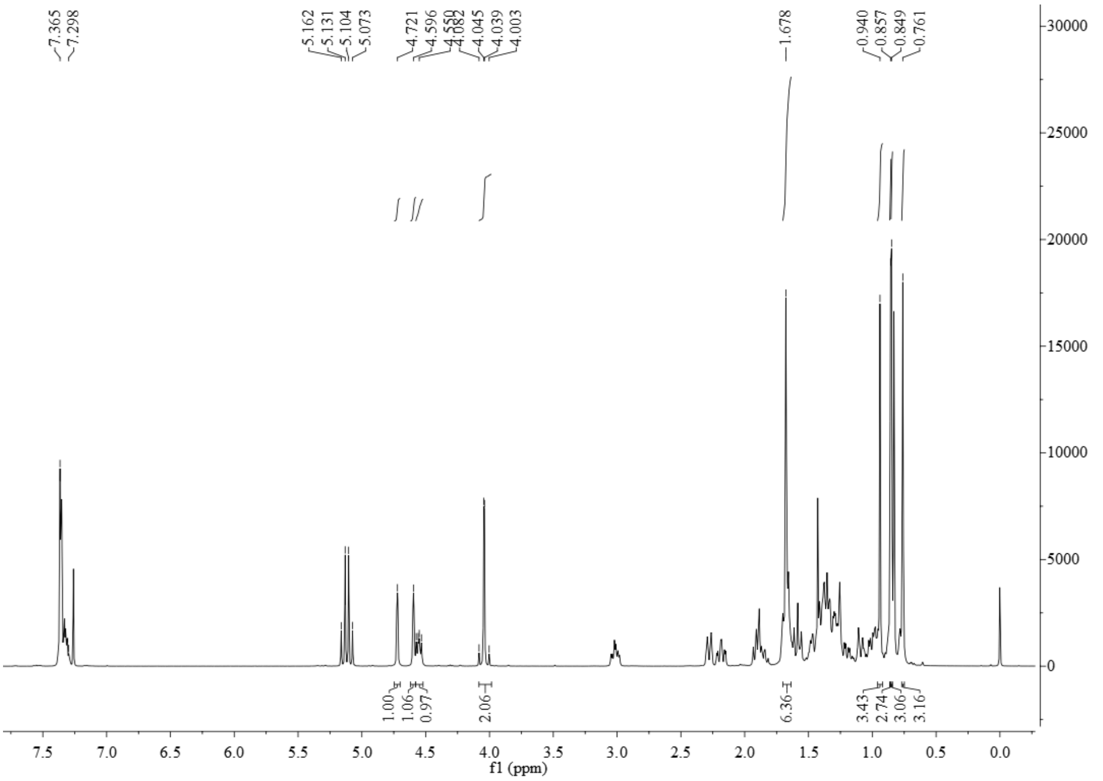

^{13}C-NMR spectra of Compound **10**

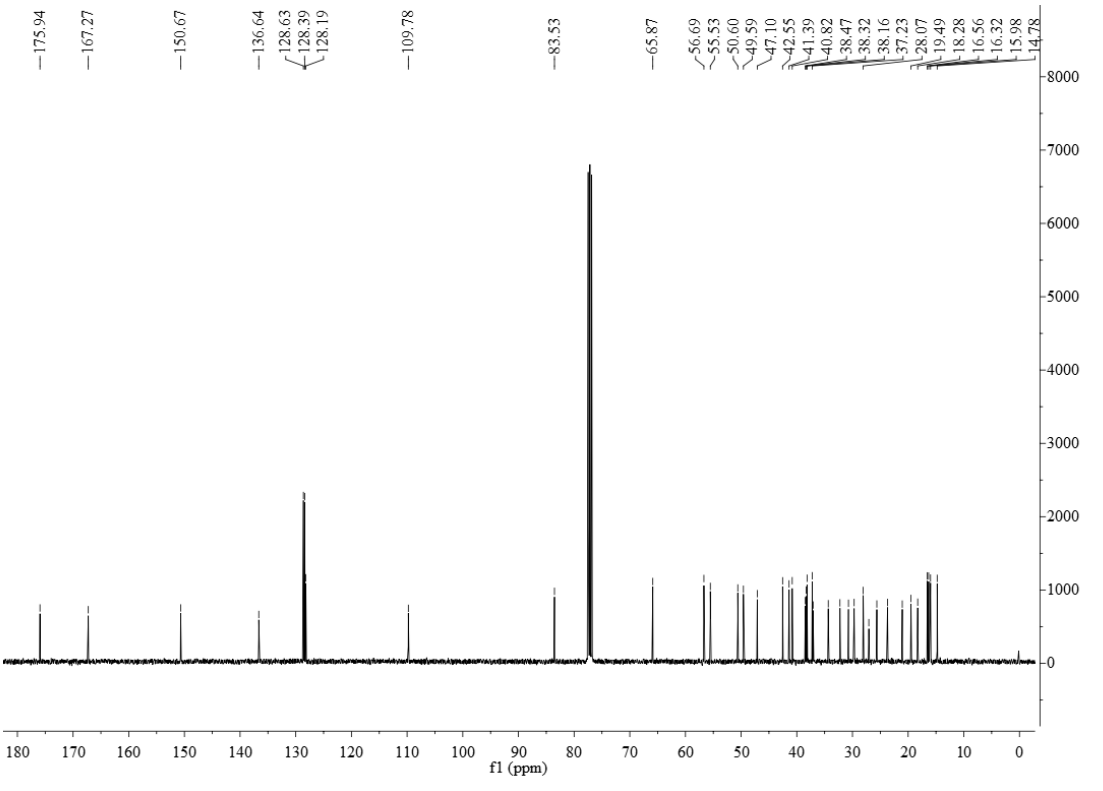

Compound 11a

^{1}H-NMR spectra of Compound **11a**

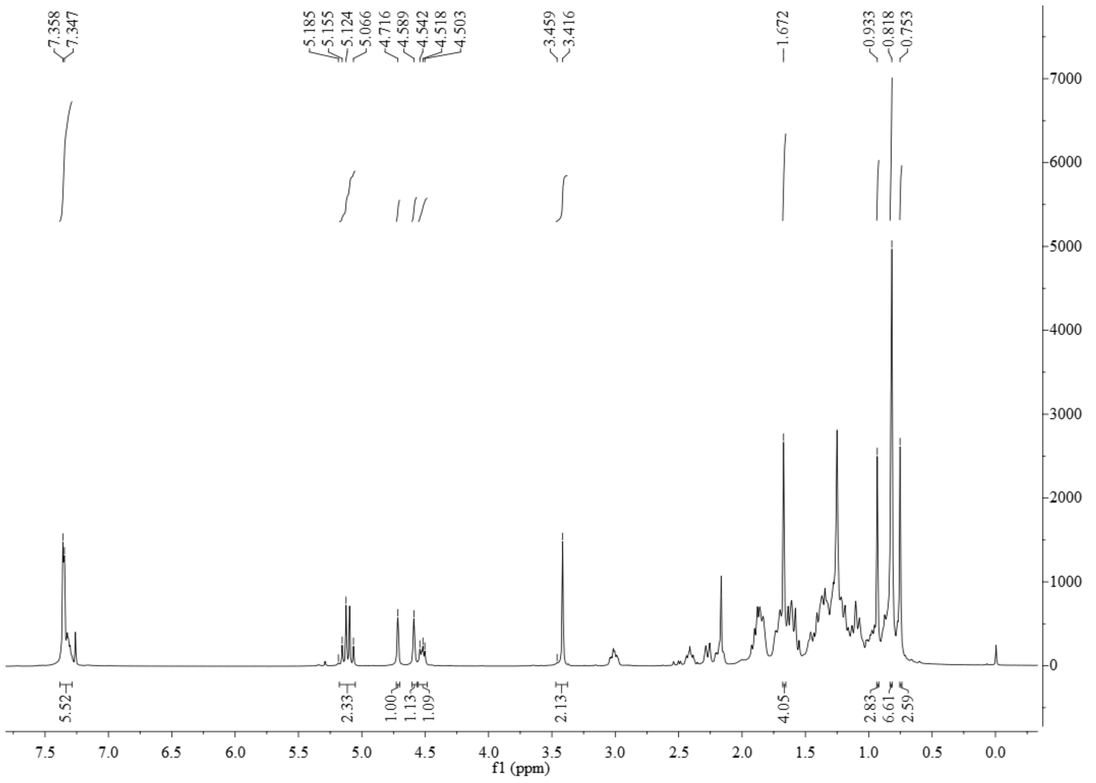

^{13}C-NMR spectra of Compound **11a**

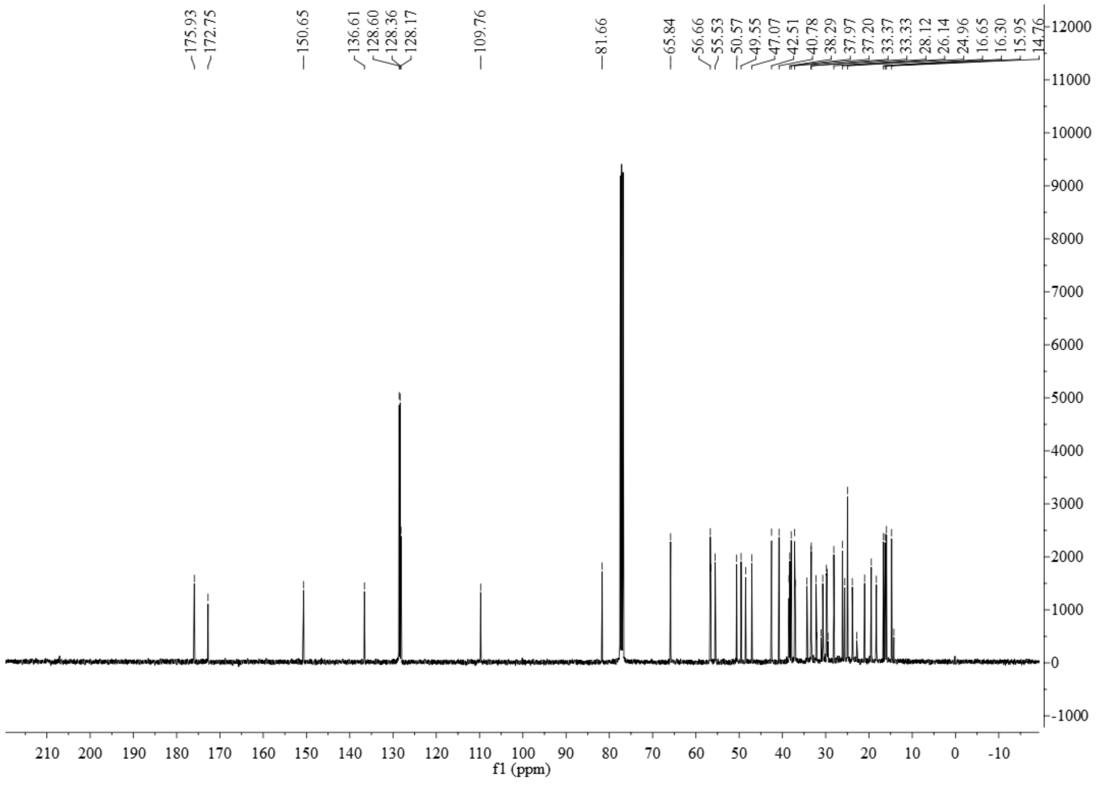

Compound 11b

^{1}H-NMR spectra of Compound **11b**

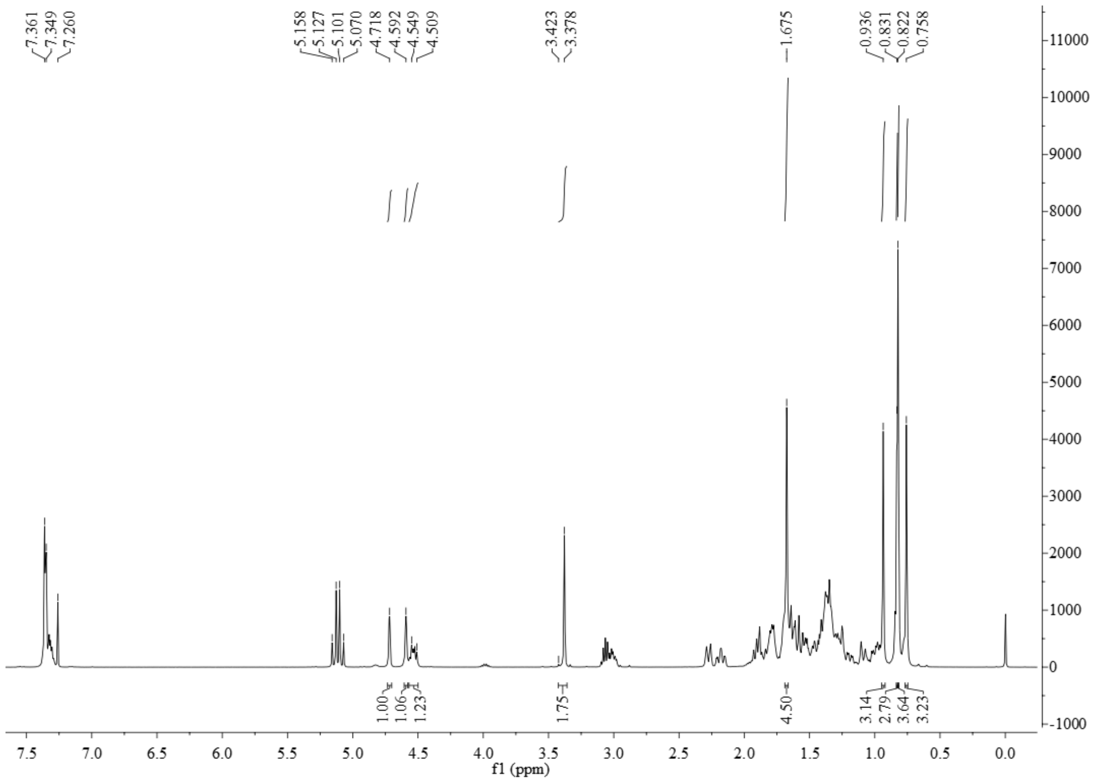

^{13}C-NMR spectra of Compound **11b**

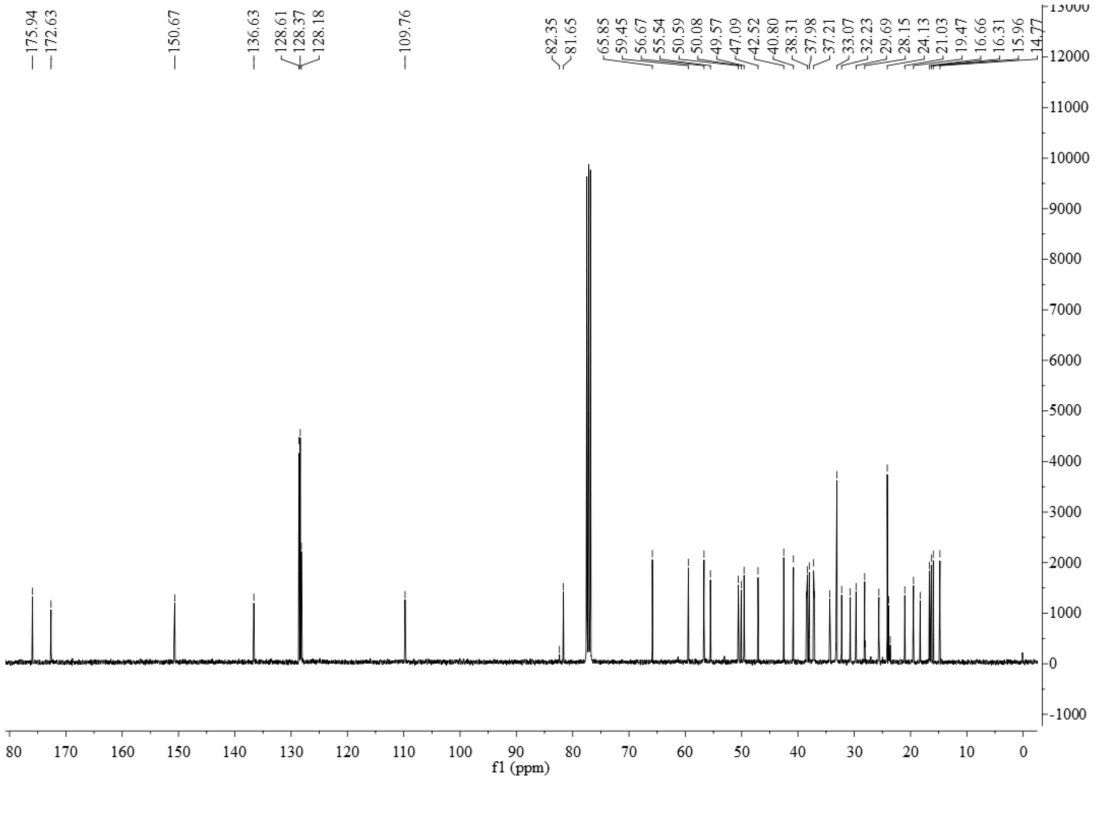

Compound 11c

^{1}H-NMR spectra of Compound **11c**

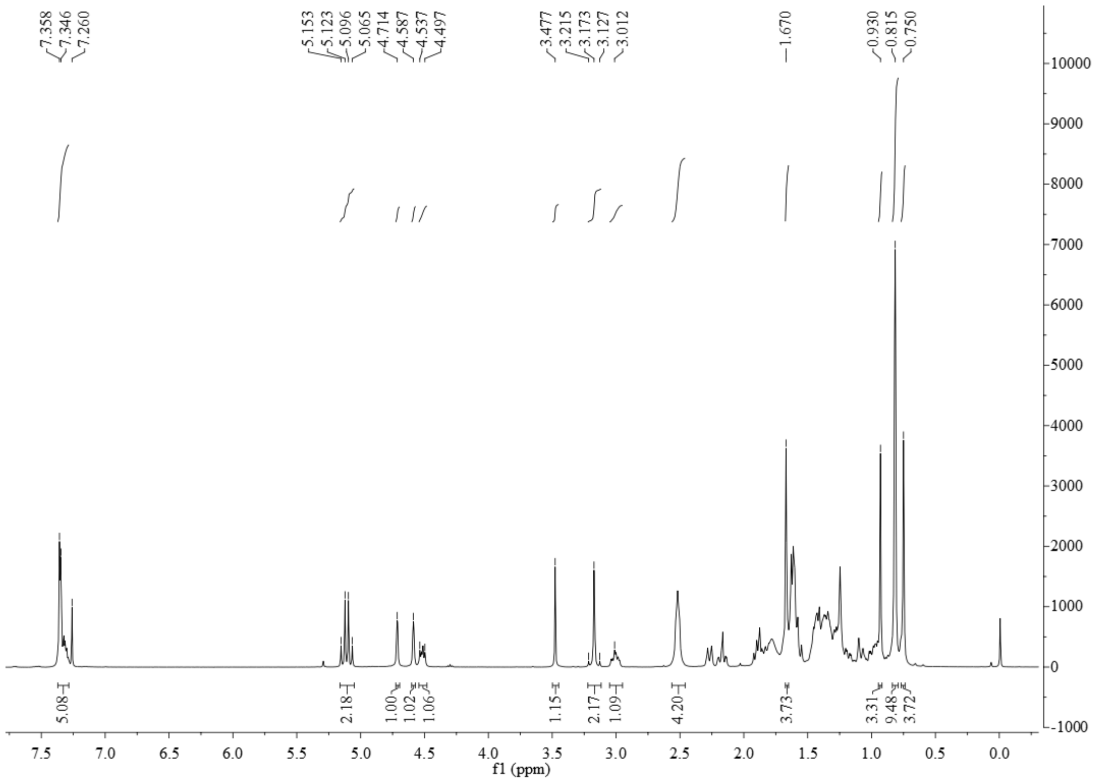

^{13}C-NMR spectra of Compound **11c**

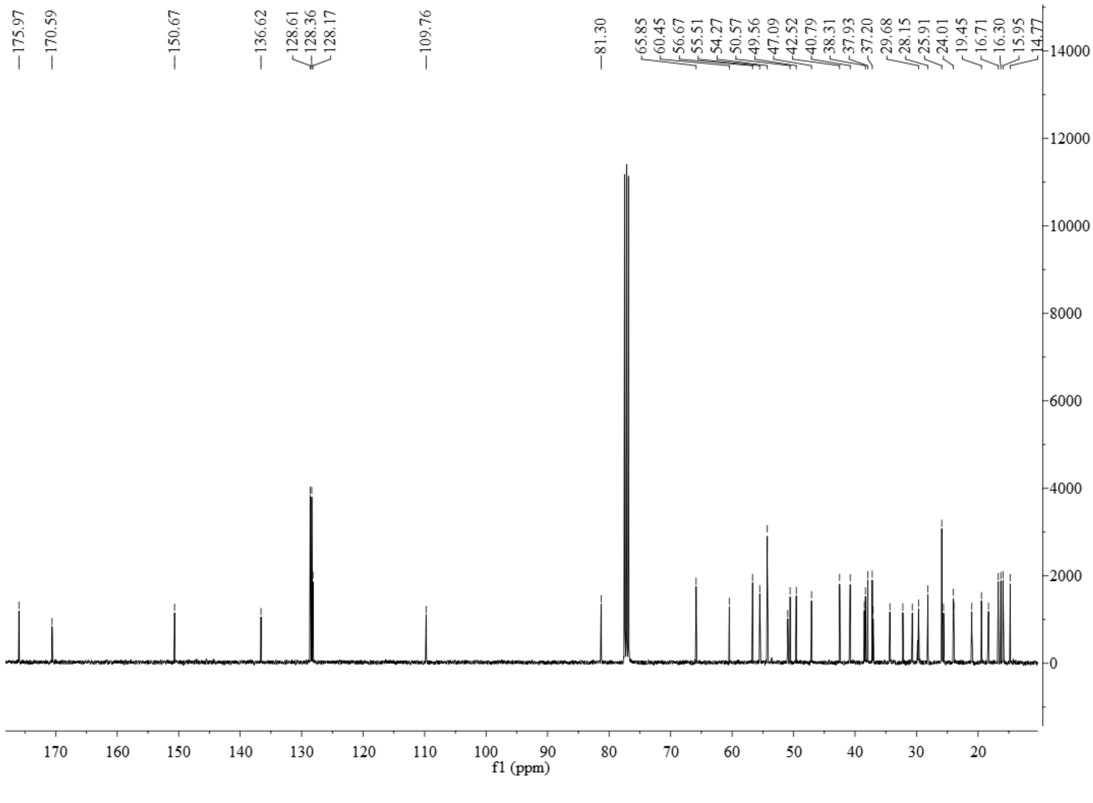

Compound 11d

^{1}H-NMR spectra of Compound **11d**

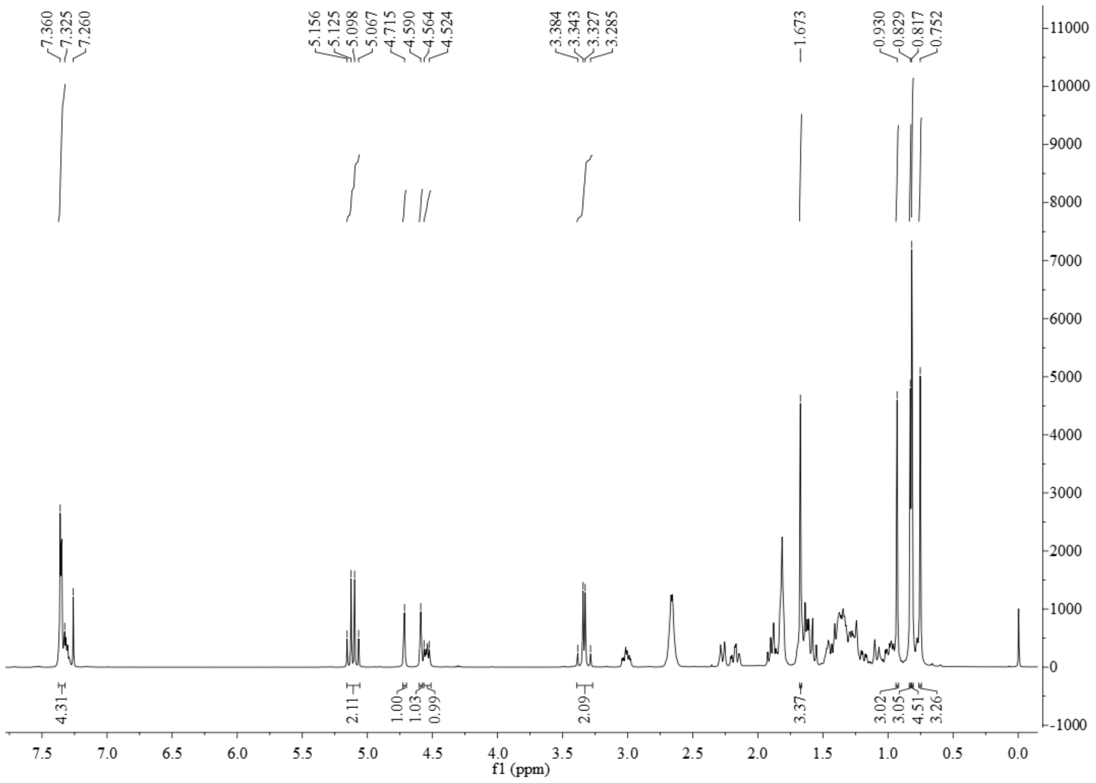

^{13}C-NMR spectra of Compound **11d**

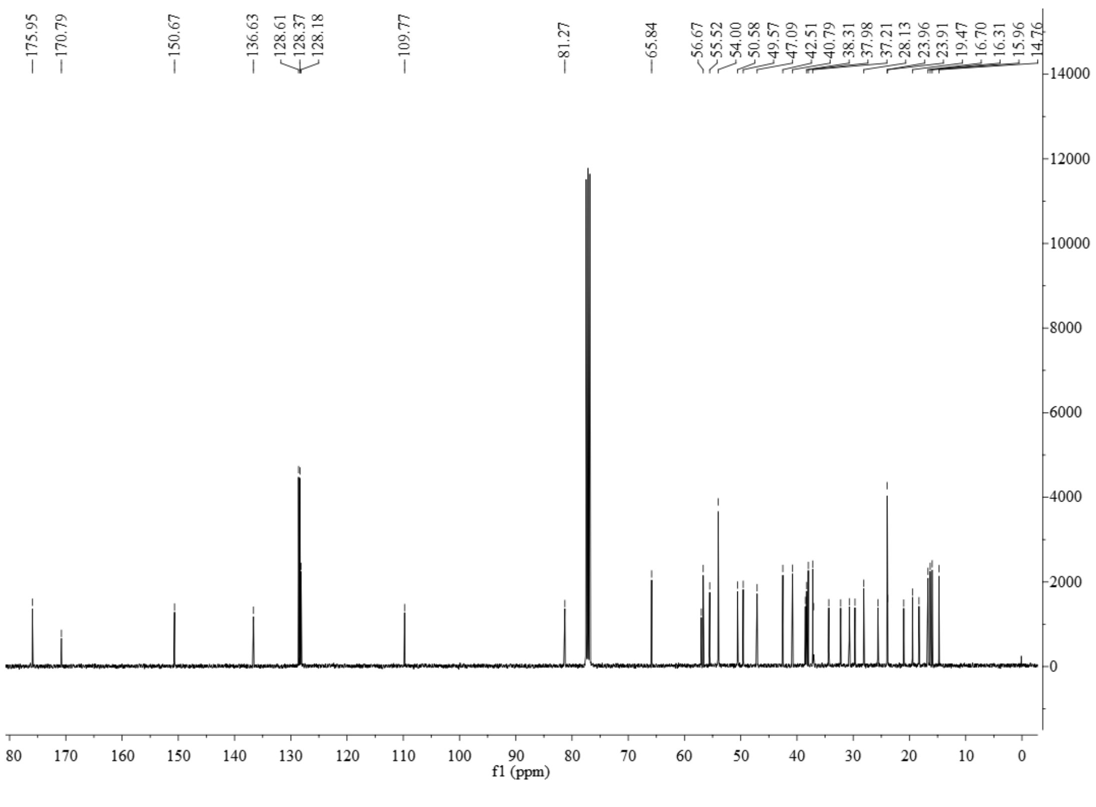

Compound 11e

^{1}H-NMR spectra of Compound **11e**

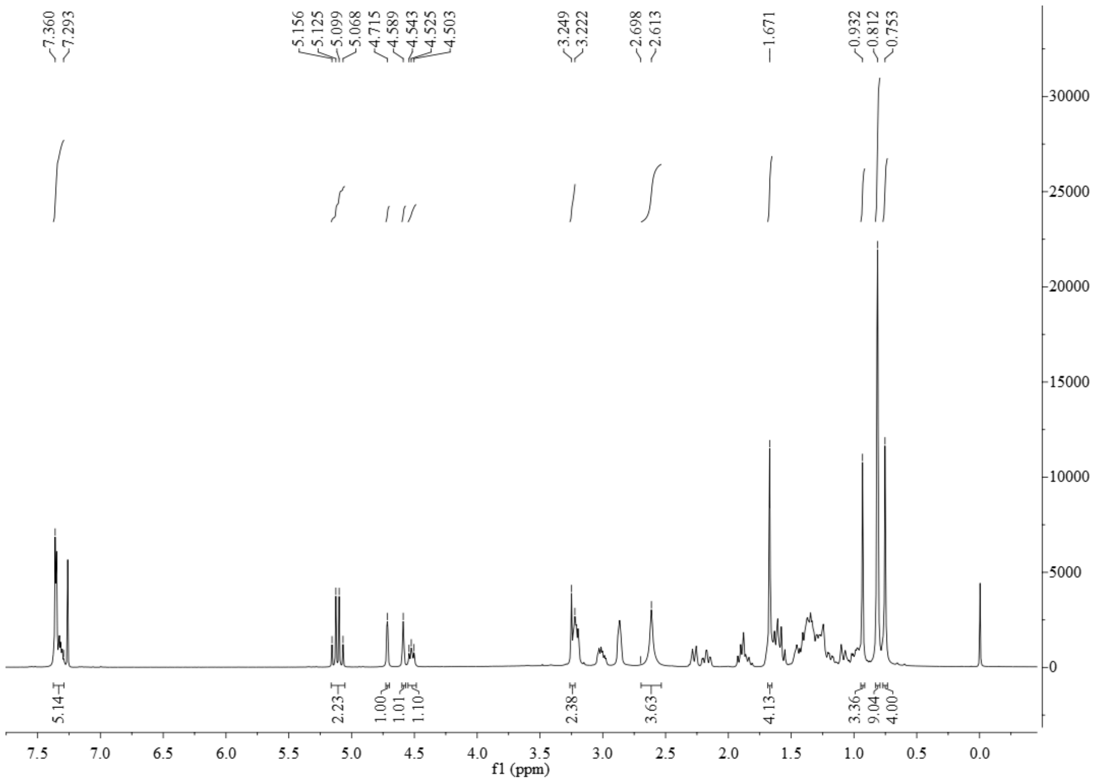

^{13}C-NMR spectra of Compound **11e**

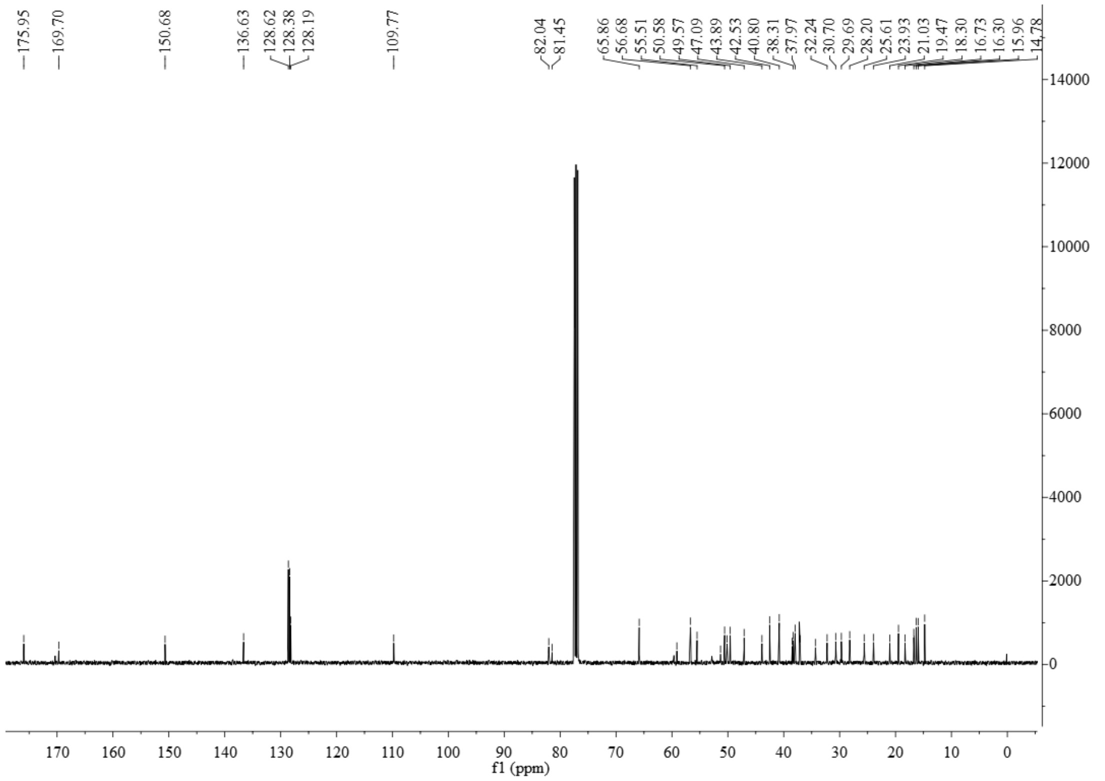

Supplement: Supplementary file 1 [file molecules-25-00948-s001.pdf]
